# Nebulised surface-active hybrid nanoparticles of voriconazole for pulmonary Aspergillosis demonstrate clathrin-mediated cellular uptake, improved antifungal efficacy and lung retention

**DOI:** 10.1186/s12951-020-00731-1

**Published:** 2021-01-11

**Authors:** Ranjot Kaur, Sarah R. Dennison, Andrea J. Burrow, Shivaprakash M. Rudramurthy, Rajan Swami, Varun Gorki, O. P. Katare, Anupama Kaushik, Bhupinder Singh, Kamalinder K. Singh

**Affiliations:** 1 University Institute of Pharmaceutical Sciences, UGC Centre of Advanced Studies, Panjab University, Chandigarh, 160 014 India; 2 School of Pharmacy and Biomedical Sciences, Faculty of Clinical and Biomedical Sciences, University of Central Lancashire, Preston, PR1 2HE UK; 3 Postgraduate Institute of Medical Education and Research, Chandigarh, 60 012 India; 4 Department of Zoology, Panjab University, Chandigarh, India 160 014; 5 Dr SSB University Institute Chem Engineering and Technology, Panjab University, Chandigarh, India 160 014; 6 UGC Centre for Excellence in Nano-Biomedical Applications, University Institute of Pharmaceutical Sciences, Panjab University, Chandigarh, 160 014 India; 7 UCLan Research Centre for Smarts Materials, University of Central Lancashire, Preston, PR1 2HE UK; 8 UCLan Research Centre for Drug Design and Development, University of Central Lancashire, Preston, PR1 2HE UK

**Keywords:** Pulmonary aspergillosis, Voriconazole, Inhalation, Antifungal, Hybrid nanoparticles, Phospholipid, Chitosan, Cell uptake, Lung retention

## Abstract

**Background:**

Incidence of pulmonary aspergillosis is rising worldwide, owing to an increased population of immunocompromised patients. Notable potential of the pulmonary route has been witnessed in antifungal delivery due to distinct advantages of direct lung targeting and first-pass evasion. The current research reports biomimetic surface-active lipid-polymer hybrid (LPH) nanoparticles (NPs) of voriconazole, employing lung-specific lipid, *i.e.*, dipalmitoylphosphatidylcholine and natural biodegradable polymer, *i.e.,* chitosan, to augment its pulmonary deposition and retention, following nebulization.

**Results:**

The developed nanosystem exhibited a particle size in the range of 228–255 nm and drug entrapment of 45–54.8%. Nebulized microdroplet characterization of NPs dispersion revealed a mean diameter of  ≤ 5 μm, corroborating its deep lung deposition potential as determined by next-generation impactor studies. Biophysical interaction of LPH NPs with lipid-monolayers indicated their surface-active potential and ease of intercalation into the pulmonary surfactant membrane at the air-lung interface. Cellular viability and uptake studies demonstrated their cytocompatibility and time-and concentration-dependent uptake in lung-epithelial A549 and Calu-3 cells with clathrin-mediated internalization. Transepithelial electrical resistance experiments established their ability to penetrate tight airway Calu-3 monolayers. Antifungal studies on laboratory strains and clinical isolates depicted their superior efficacy against Aspergillus species. Pharmacokinetic studies revealed nearly 5-, 4- and threefolds enhancement in lung AUC, T_max_, and MRT values, construing significant drug access and retention in lungs.

**Conclusions:**

Nebulized LPH NPs were observed as a promising solution to provide effective and safe therapy for the management of pulmonary aspergillosis infection with improved patient compliance and avoidance of systemic side-effects. 
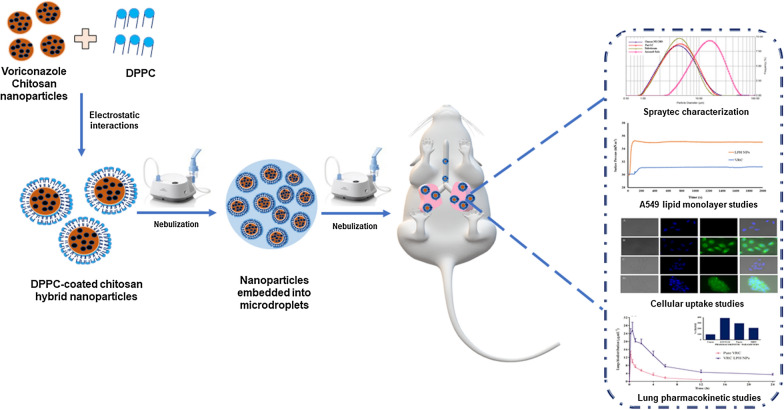

## Introduction

Pulmonary aspergillosis (PAP) has raised vital concerns in clinical healthcare, as it mainly affects people with weak immune systems. It is one of the major co-morbid infections in diseases like asthma, influenza, chronic obstructive pulmonary disease, tuberculosis, cancer, etc. [1, 2]. Recent reports on Covid-19 patients have also shown PAP as one of the major infections associated in almost 30–35% of the cases on mechanical ventilators, thus increasing their mortality rate by as much as 50% [3–6]. Primarily caused by the ubiquitous fungus, *i.e., Aspergillus fumigatus,* the leading risk factors associated with this opportunistic infection includes damaged respiratory epithelium, impaired mucociliary clearance and attenuated local immune system [7].

The currently available antifungal regimen is restricted to oral and parenteral routes only, which limits the therapeutic availability of drugs in the lungs owing to limitations of poor solubility, low permeability and rapid clearance of drug [8]. Pulmonary route offers myriad advantages like faster onset of action, localized non-invasive therapy, circumnavigation of the first-pass effect and reduced systemic side effects [9]. Concerted and immediate research efforts, therefore, are required to develop an effective pulmonary drug delivery system for the therapeutic management of PAP. In the absence of any of such commercially available product, the nebulized liposomal amphotericin B (AmB) has been administered as an inhalation for off-label use [10]. Also, in mechanically ventilated Covid 19 patients, nebulised liposomal AmB has been successfully given as a prophylactic therapy to avoid any potentially detrimental outcomes [3]. However, AmB usage needs to be restricted owing to its nephrotoxic nature [11]. Voriconazole, a first-line antifungal triazole, is strongly recommended as the preferred antifungal triazoles in the therapeutic and prophylactic management of PAP, as per the guidelines of the Society of Infectious Diseases Pharmacists [12, 13]. Voriconazole, administered through conventional routes, has demonstrated equivalent or superior efficacy to AmB with reduced side-effects in various clinical trials [14, 15]. Not much, however, is known regarding its pulmonary safety, efficacy and pharmacokinetics, upon nebulization. Extensive investigations, therefore, are called for to ratify the effectiveness of voriconazole through nebulization in the potential treatment of PAP.

Recent advancements in the realm of nanotechnology have provided significant impetus to inhalation drug delivery. Drug nanocarriers tend to provide definitive benefits of superior biopharmaceutical properties, enhanced lung permeability, and improved dissolution profiles vis-à-vis the corresponding conventional therapy [16–19]. A therapeutic efficacy of the inhaled nanoparticles is highly dependent on their ability to overcome extrapulmonary and intracellular pulmonary barriers. Therefore, designing nanocarriers with an aim to minimize their premature mucociliary clearances and improving internalization at the air-lung interface through positive bio-interactions, has been gaining continued importance [16, 20]. This rationalizes the burgeoning interest in the application of bioinspired materials for fabricating nanoparticles for pulmonary delivery [21, 22].

**Scheme 1:**
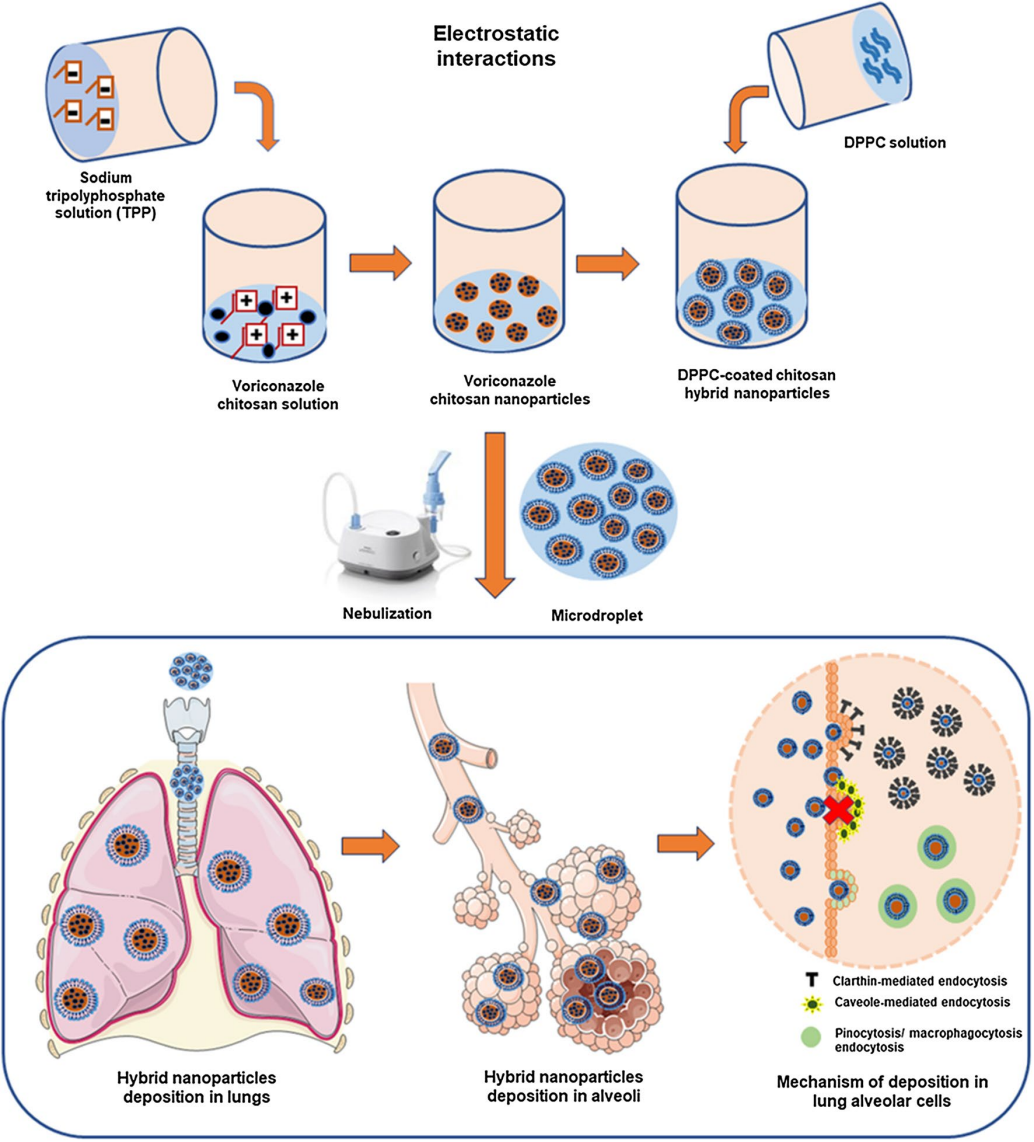
Preparation of dipalmitoyl phosphatidylcholine (DPPC) coated chitosan hybrid nanoparticles by electrostatic interaction method. The nanoparticle dispersion was nebulised as microdroplets for effective targeting the respiratory airways by the mechanism of diffusion. Once deposited, their cellular uptake inside the lung epithelial cells is a determinant of their particle size and surface charge

Pulmonary surfactant being the first biological barrier of the respiratory surface, restricts the entry of foreign particles into lungs [23, 24]. Native pulmonary surfactant consists of various lipids (circa 90%) and surfactant proteins (circa 10%). Amongst the lipids, dipalmitoylphosphatidylcholine (DPPC), a lung-specific phospholipid, constitutes as the major portion (circa 50–60%) of the lung surfactant [23]. Accordingly, we herein investigate nebulized DPPC coated chitosan (CH) nanoparticles (NPs) for the pulmonary delivery of voriconazole. CH, a natural polysaccharide, has shown immense potential and acceptance in the inhalational drug delivery due to its biodegradable, mucoadhesive, permeation-enhancing and controlled release characteristics [25–27]. The presence of DPPC coating on NPs surface enhance its biocompatibility as well as increase its interaction with the pulmonary surfactant monolayers (PSM), which line the inner epithelial surface of lung alveoli at the air–liquid interface [21, 23]. Moreover, it minimizes the macrophage uptake, thereby facilitating NPs diffusion and internalization into the lung epithelial cells. These surface-active lipid-polymer hybrid (LPH) nanosystems, with distinctive advantages of the polymeric as well as the lipidic systems, has shown promise for delivering voriconazole to the lungs. The present work encompasses development of LPH NPs, following the systematic principles of Quality by design (QbD), and subsequent extensive characterization and evaluation, including aerosol characterization (Scheme 1). Besides, the present studies also examined the air-lung lipid membrane interaction via Langmuir–Blodgett trough and cellular transport pathways of NPs, using fluorescence microscopy and flow cytometric analysis. Furthermore, the antifungal efficacy of NPs was assessed in laboratory strains as well as clinical respiratory isolates of the *Aspergillus* species. Comprehensive in vivo studies to determine the lung and plasma pharmacokinetics, and to establish the safety of the formulation unearthed the immense potential of these hybrid systems, in the domain of pulmonary drug delivery of antifungals.

## Materials and methods

Voriconazole and DPPC were gifted by M/s Life Care Laboratories Ltd., India, and by M/s Lipoid, Germany, respectively. Medium-molecular weight CH and Rhodamine 123 (Rh-123) were purchased from M/s Sigma Aldrich, UK, while sodium tripolyphosphate (TPP) and Tween 80 (T80) were procured from M/s Acros Organics and M/s Fisher Scientific, India, respectively. All other chemicals, reagents, and solvents employed during the current research work were of analytical grade and were used as acquired. Adenocarcinoma human alveolar basal epithelial (A549) cells and human bronchial epithelial (Calu 3) cells were purchased and maintained as per M/s ATCC, Manassas, USA.

### Synthesis of chitosan nanoparticles (CH NPs)

CH NPs were prepared by ionic gelation method [28]. Briefly, CH was added to 1% (v/v) acetic acid solution and stirred for overnight on a magnetic stirrer at 500 rpm. An anionic cross-linker (*i.e.*, TPP in the ratio of 1: 2.5 v/v) was added to CH solution containing T80. Similarly, the drug- or dye-loaded NPs were prepared by dissolving 20 mg of voriconazole, or 10 µg/mL of Rh-123, in 2 mL of ethanol and then adding to the CH-surfactant solution, before drop-wise addition of a suitable cross-linker.

### Factor screening and optimization as per design of experiments (DoE)

Factor screening studies were conducted employing a seven-variable eight-run Taguchi design to identify the highly influential product and process variables affecting CH NPs (Additional file 1: Table S1**)**. Half-normal and Pareto plots were used for quantitative analysis of the influence of all the individual product and process variables on each of the selected critical quality attributes (CQAs) [29]. A Box-Behnken design (BBD) with 17 experimental runs (Additional file 1: Table S2) was employed for optimization of CH NPs using Design Expert^®^ software version 11.0 (M/s Stat-Ease, USA) [29, 30].

### Preparation of lipid-polymer hybrid nanoparticles (LPH NPs)

LPH NPs were prepared by the electrostatic interaction method [31]. Briefly, different ratios of CH NPs dispersion and ethanolic solution of DPPC (2 mL) were incubated for 20 min at 4 °C and subjected to high energy sonication employing a probe sonicator (M/s Vibra Cell Sonics, USA) for 5 min at 30% amplitude for coating a lipid layer on the CH NPs. The optimized LPH NPs were subsequently lyophilized, employing a suitable combination of cryoprotectants.

### Characterization studies

The particle size (PS), zeta potential (ZP), and polydispersity index (PDI) of the NPs were analyzed using dynamic laser scattering (DLS) equipment (Nano ZS, M/s Malvern, UK), set at a temperature of 25 °C. The nanoparticle dispersion was diluted 50-folds with purified water, prior to each such measurement.

Entrapment efficiency (EE) was determined by both indirect and direct methods. LPH NPs (1 mL) were taken and centrifuged at 20,000*g* for 2 h at 10 °C. For indirect method, supernatant was removed and was analyzed for free VRC content, employing a previously validated high-performance liquid chromatography (HPLC) method on a chromatograph (M/s Waters, USA) with acetonitrile and acetic acid solution (50:50) as mobile phase, at flow rate of 1 mL.minute^−1^ [32, 33]. Further, the direct method involved the washing of pellet, thrice with water, and then dissolving it in 2% acetic acid solution and methanol, followed by probe sonication for 15 min and filtration through 0.4-micron syringe filter. Subsequently, VRC content was analyzed using previously validated HPLC method. The surface morphology of voriconazole and NPs were investigated employing a Field Emission Scanning Electron Microscope (FESEM, Su8010, M/s Hitachi, Japan). Further, the drug-excipient compatibility studies were carried out by KBr pellet method using a Fourier Transformed Infra-Red (FTIR) spectrometer (M/s Perkin Elmer, USA). Spectra for VRC, CH, TPP and DPPC, along with their physical mixture (P_mix_) and LPH NPs, were recorded and studied for any noticeable shift(s) in the peaks. Crystallographic structure of the pure drug, physical mixture, and LPH NPs were recorded on a powder X-ray diffractometer (M/s PAN analytical, Netherlands). The samples were packed in sample holder and scanned between 5 and 50° in 2θ [34].

### In vitro drug release studies

Drug release studies were performed employing the dialysis sac technique [35]. The NPs dispersion (containing VRC equivalent to 1.4 mg) or voriconazole suspension were placed in a dialysis sac (12 kDa, M/s Sigma Aldrich, UK), tethered at both the ends and suspended at 37 ± 0.5 °C at 100 rpm in 20 mL of phosphate buffer saline (PBS 7.4) and 0.1% of Tween 80 (v/v) to maintain the desired sink conditions. Samples were periodically withdrawn and assayed for voriconazole content using HPLC. Drug release profiles of LPH NPs were plotted and underlying drug release kinetic behaviour was ascertained [36].

### Microdroplet laser diffraction analysis

Spraytec laser diffraction equipment (M/s Malvern Panalytical, Malvern, UK) was employed to acquire the volume droplet size distribution of the nebulized aerosol-cloud [37]. The instrument was equipped with He–Ne laser to acquire the volume-droplet size distribution of an aerosol cloud instantaneously, with an acquisition frequency of 10 kHz. [38]. Four types of nebulizers, three of these the jet nebulizers, viz, Omron C803 (Omron), Pari LC (Pari), and Sidestream (Respironics), and one mesh nebulizer viz, Aeroneb (Aerogen), were used to generate microdroplets of LPH NPs dispersion. The experiment was performed in triplicate, and the parameters, such as Dx (10), Dx (50), and Dx (90), were calculated employing Spraytec version 3.20 software. The nebulizer delivering the dispersions with best volume mean diameter (VMD) was selected for further particle deposition studies.

### Next-generation impactor (NGI) studies

Particle deposition studies of LPH NPs dispersion were performed employing an NGI at a flow rate of 15 L. minute^−1^. Sidestream nebulizer was attached to the induction port of an NGI (M/s Copley Scientific, UK) using a mouthpiece adapter and filled with 10 mL of LPH NPs dispersion with an aerosol collection time of 5 min [39]. After completing the measurement, the cup holder tray was taken out, and each stage of the impactor was washed with 10 mL of extraction solvent (methanol and 1% acetic acid solution), filtered and subjected to HPLC analysis for determining the voriconazole content. Mass median aerodynamic diameter (MMAD) and geometric standard deviation (GSD) were computed employing online MMAD calculation software [40]. Other parameters, like fine particle fraction (FPF) and emitted dose (ED), were also calculated as per the standard formulas [41].

### Influence of nebulization on the particle size of LPH NPs

Nebulizers generate extensive shear forces on the particles getting aerosolized, which could alter their integrity during the process. In order to ensure that the integrity and stability of NPs is maintained during nebulization, the PS of the LPH NPs was examined, before and 20 min after nebulization using DLS [42].

### Surface activity using langmuir troughs

#### Surface activity determination of drug and LPH NPs

Surface activity of native voriconazole solution (1 × PBS pH 7.4) and drug loaded LPH NPs was investigated using a 15 cm^2^ Teflon Langmuir trough (M/s Biolin Scientific, UK). Samples were injected into 10 mM, pH 7.4 PBS using a Hamilton microsyringe to yield the final drug concentrations ranging between 0 and 100 µM. Surface pressure changes were monitored by the Whilemly plate (Whatman’s CH1 filter paper) attached with a microbalance [43] and were plotted against drug concentrations.

#### Interaction of LPH NPs with model cell membrane monolayers

Potential interaction of LPH NPs and voriconazole solution (1 × PBS 7.4) at the air-lung lipid membrane interface was investigated using lung cell monolayer mimics of either DPPC or cellular lipids extracted from A549 cells as described in Additional file section [44]. Biophysical interaction of LPH NPs and voriconazole solution with lipid monolayer was studied at a constant pressure using 80 mL of 601 M Teflon trough with movable barriers. Chloroform solution of the lipids (0.5 mM) was spread onto a buffer subphase (1x PBS pH 7.4), and the solvent was allowed to evaporate (30 min). The monolayer was compressed at a velocity of 10 cm^2^ min^−1^ to gain a surface pressure of 30 m N m^−1^, which is mimetic of naturally occurring membranes [45]. Using barrier control, the lipid monolayer was maintained at this area throughout the experiment. After the equilibration of monolayer for 10 min, the samples were injected separately into subphase to yield the final concentration of 50 μM. Augmentation in the surface pressure was monitored using NIMA software and was subsequently plotted against time [43].

### Antifungal efficacy

The antifungal susceptibility of voriconazole, blank and drug loaded LPH NPs was carried out by the micro broth dilution method using Roswell Park Memorial Institute 1640 as growth medium. The laboratory strains as well as the clinical isolates of *Aspergillus* viz*., Aspergillus fumigatus, Aspergillus flavus* (obtained from bronchoalveolar lavage), were exposed to LPH NPs at the drug concentrations varying between 0.029 and 14.84 µg mL^−1^. Besides, *Candida krusie* and *Candida parapsilosis* strains were also exposed to LPH NPs. The incubation for *Aspergillus* and *Candida* species was carried out at 35 °C in a dark and humid chamber for a period of 24 and 48 h, respectively. The values of MIC were determined employing a visual endpoint, as per the recommendations of the Clinical and Laboraotry Standard Institute (CLSI) [46].

### In vitro cell line studies

#### Safety studies

The cell viability profile of voriconazole, blank and drug-loaded LPH NPs was investigated on A549 (5 × 10^3^ cells/cm^2^) and Calu 3 cells (1 × 10^4^ cells/cm^2^) in a 96-well plate (M/s Thermo Fischer Scientific, Denmark) via the Presto blue assay. Cells were seeded using a standard protocol, and the culture medium was replenished with 90 µL of the medium containing LPH NPs.

(0.31 to 40 µg mL^−1^) and incubated for a period of 48 h. PrestoBlue^TM^ (M/s Invitrogen, USA) 10 µL, was added to each of the well and incubated for a period of 1 h, following which the fluorescence was recorded using a microplate reader (M/s Tecan, Männedorf, Switzerland), at the excitation and emission wavelengths of 535 and 612 nm, respectively [30].

#### Qualitative cellular internalization study

A549 (1×10^5^ cells/well) and Calu 3 (2×10^5^ cells/well) were seeded on coverslips in 6 well-plates (M/s Thermo Scientific, Denmark), and transferred to a humidified incubator. When the cells reached 85–90% confluency, the plates were taken out of the incubator, washed with PBS (0.1 M, pH 7.4, 1 mL) twice and subsequently incubated for a period of 4 h with 10 μg mL^−1^ of Rh-123 loaded-LPH NPs (Rh-LPH NPs). Following incubation, the treated cells were washed using PBS thrice and analysed under a fluorescence microscope (M/s Carl Zeiss, UK) with an oil lens at 63x magnification [30].

#### Quantitative cellular uptake studies

The cellular uptake studies were conducted to evaluate the effect of concentration and time on the uptake of Rh-LPHNPs in A549 (5×10^4^ cells/well) and Calu 3 (1x 10^5^ cells/well) cells using a flow cytometer (Guava^®^ easyCyte HT, M/s Merck, Germany). Varied concentrations of formulation (*i.e.,* 1, 2.5, 5 μg mL^−1^) were incubated at 37 ± 1 °C in pre-cultured lung epithelial cells in 12 well-plates for varying time intervals, *i.e.*, 0.5, 1, 2, 4 and 6 h, and estimated for mean fluorescence intensity [47].

#### Mechanistic endocytosis pathway analysis

A549 (5×10^4^ cells/well) and Calu 3 (1×10^5^cells/well) were seeded in 12 well-plates. Upon 80–90% confluency, the cells were exposed to different endocytosis inhibitors for 90 min before treating with Rh-LPH NPs. Sucrose (0.45 M), nystatin (5 μg mL^−1^), and cytochalasin B (5 μg mL^−1^) were employed to block the clathrin-mediated, caveolae/lipid rafts and macropinocytosis pathways, respectively. After 90 min, the medium containing endocytic inhibitors was removed and replaced with 1 μg mL^−1^ of Rh-LPH NPs, diluted with medium-plus endocytic inhibitors, followed by incubation for 4 h at 37 °C. The effect of energy-dependent endocytosis was also observed by placing the cells at 4 °C. Samples were processed, as per the standard protocol, and subjected to flow cytometer [47].

#### Permeability assay

An aliquot of 400 μL of Calu-3 cell suspension (5×10^5^ cells/cm^2^) was seeded on the apical side of the Transwells^®^ (0.4 mm pore size, 12 mm diameter), and 1 mL of complete medium was added to the basolateral side to allow the monolayer to grow at the liquid-liquid interface (LLI). The transepithelial electrical resistance (TEER) of the Calu-3 monolayers was monitored at the pre-determined day intervals using a volt-ohm meter (Millicell ERS-2, M/s Merck, Darmstadt, Germany) and the inserts showing TEER value less than 1000-ohm cm^−2^ were excluded from the study. Moreover, the cell monolayer integrity was also challenged by the application of highly permeable azo dye, *i.e.,* Evans blue (10 μg mL^−1^). After confirming the monolayer integrity, the medium from the apical side was replaced with Rh-LPH NPs (1 μg mL^−1^). Aliquots of samples (100 μL each) were withdrawn from the basolateral side after the periodic time intervals of 0.5, 1, 2, 4, and 6 h, and subsequently replaced each time with fresh transport medium [48]. The samples were analyzed using a fluorescence microplate reader (GENios Pro^®^, M/s Tecan UK Ltd., Theale, UK) to monitor the concentration of the Rh-LPH NPs, while setting the excitation wavelength at 535 and emission wavelength at 612 nm. A plot between the cumulative amount of Rh-LPH NPs permeated and time was constructed and apparent permeability (Papp) was computed using Eq. 1 [30].

1 $${\text{Papp}} = \left( {\frac{1}{{{\text{A}} \times {\text{Co}}}}} \right) \times \left( {\frac{{{\text{dQ}}}}{{{\text{dt}}}}} \right)$$

where, A is the surface area of membrane (1.12 cm^2^), C_0_ is the initial Rh-LPH NPs concentration on apical side (1 µg mL^−1^) and dQ/dt is the steady state flux.

### Lung and plasma pharmacokinetic studies

In vivo studies were carried out on Balb/c mice (23 ± 2 g) as per ethical approval from the Institutional Animal Ethics Committee of the Panjab University (PU/45/99/CPSEA/IAEC/2019/243). The mice were kept in the open polycarbonate cages associated with wire-lid holding chow-and-water bottles, in a well-ventilated room at a temperature of 25 ± 2 °C and humidity of 65–70%. Animals were fed with standard pellet diet and water ad libitum. The mice were acclimatized for a period of 14 days prior to animal experimentation. A total of 48 animals used for the study were divided into two groups in a randomized manner. All the animals were fasted for over-night prior to experimentation [49]. Group I received LPH NPs inhalation, while Group II received inhalation of a commercially available intravenous formulation of voriconazole (Vorier, M/s Zydus Cadila, India), at a dose of 15 mg with the flow rate of 1.40 ± 0.13 L min^−1^ [50]. The time period of 20 min was chosen for nebulization in line with usual time opted in clinical practice [51]. Mice (n = 3 each) were euthanized by cardiac puncture at specified time intervals of 0.2, 0.5, 1.0, 2.0, 4.0, 8.0, 12.0 and 24.0 h [52]. Whole blood samples were collected in the heparinized vials, centrifuged for 10 min at 10,000 rpm, and the resultant plasma samples were stored at −20 °C, till further analysed. Further, lungs were excised and stored at −20 °C till quantitatively analysed for voriconazole content employing HPLC [32]. Different pharmacokinetic parameters, like maximum lung and plasma concentrations (*i.e.*, C_max_) and the corresponding time points to reach C_max_ (*i.e.,* T_max_), area under the curve (*i.e.,* AUC), and mean residence time (*i.e.,* MRT), were computed employing PK solver spreadsheet software [53].

### In vivo safety studies

The safety studies for LPH NPs were conducted in Balb/c mice on four of the animal groups. Group I was designated as naïve group, while Groups II, III, and IV received nebulization of normal saline (NS 0.9%), voriconazole (15 mg), and LPH NPs (equivalent to 15 mg of voriconazole), respectively, at a flow rate of 1.40 ± 0.13 L min^−1^. Treatments were administered regularly twice-a-day for 20 min, consecutively for a period of 21 days. The animals were sacrificed at the end of 21 days-period by cardiac puncture [50], and various tissue specimens were examined for any noticeable morphological changes.

### Stability studies

The stability of the lyophilized voriconazole LPH NPs was determined employing humidity chambers (M/s Newtronic Lifecare Equipment, Mumbai, India). The samples were stored in glass vials for a period of 90 days at different temperature and humidity conditions, *i.e.,* 5 °C ± 3 °C, 25 °C ± 2 °C/60 ± 5% RH; 40 °C ± 2 °C/75 ± 5% RH [54]. The lyophilized samples were reconstituted in sterile water at 0, 30 and 90 days, bath-sonicated for 30 min, and were analysed for possible change in PS, PDI and EE.

### Statistical data analysis

Statistical analysis was conducted by one-way analysis of variance (ANOVA), and an unpaired *t* test was used for individual comparisons employing Prism Software (M/s GraphPad Inc., La Jolla, USA).

## Results

### DoE-based screening and optimization studies

Considered as a vital prioritization exercise as a prelude to optimization studies, factor screening assumes factor additivity and absence of any interaction(s), thus calling for employment of an apt linear model (Eq. 2). Analysis of consequent half-normal and Pareto plots unravel that CH, TPP, and T80 form as highly influential CMAs for CH NPs formulation, as is apparent from their statistically significant (p < 0.05–0.001) effects, much above the critical t-value and Bonferroni lines (Additional file 1: Figure S1). Other factors, such as pH (4.0), stirring speed (800 rpm), stirring time (60 min), and probe sonication time (3 min), were accordingly kept as constant.

2 $$y\;\text{ = }\;\beta_{0} \;\text{ + }\;\beta_{1} X_{1} \;\text{ + }\;\beta_{2} X_{2} \;\text{ + }\;\beta_{3} X_{3} \;\text{ + }\;\beta_{4} X_{4} \;\text{ + }\;\beta_{5} X_{5} \;\text{ + }\;\beta_{6} X_{6} \;\text{ + }\;\beta_{7} X_{7}$$

where y is the response variable, β_0_ represents the intercept form, and β_1_ to β_7_ depict the coefficients of the corresponding linear model terms.

The response surface optimization graphs tend to facilitate explicit comprehension of the studied factors, along with their possible interactions, of each of the investigated CQAs of the CH NPs. The 3D-and 2D response surface plots were constructed for each of the CQAs, viz. PS, PDI, ZP, EE, and DL (Additional file 1: Fig. S2 (a-o). The magnitudes of coefficients of the second-order polynomial equation are provided in Additional file 1: Table S3. Additional file 1: Figure S3 illustrates the overlay plot, with design space marked in yellow colour and values of the CQAs obtained for the optimized formulation of CH NPs, comprising of CH (2.1 mg mL^−1^), TPP (1.9 mg mL^−1^) and T80 (3.6 mg mL^−1^) [29].

### Characterization studies

**Fig. 1:**
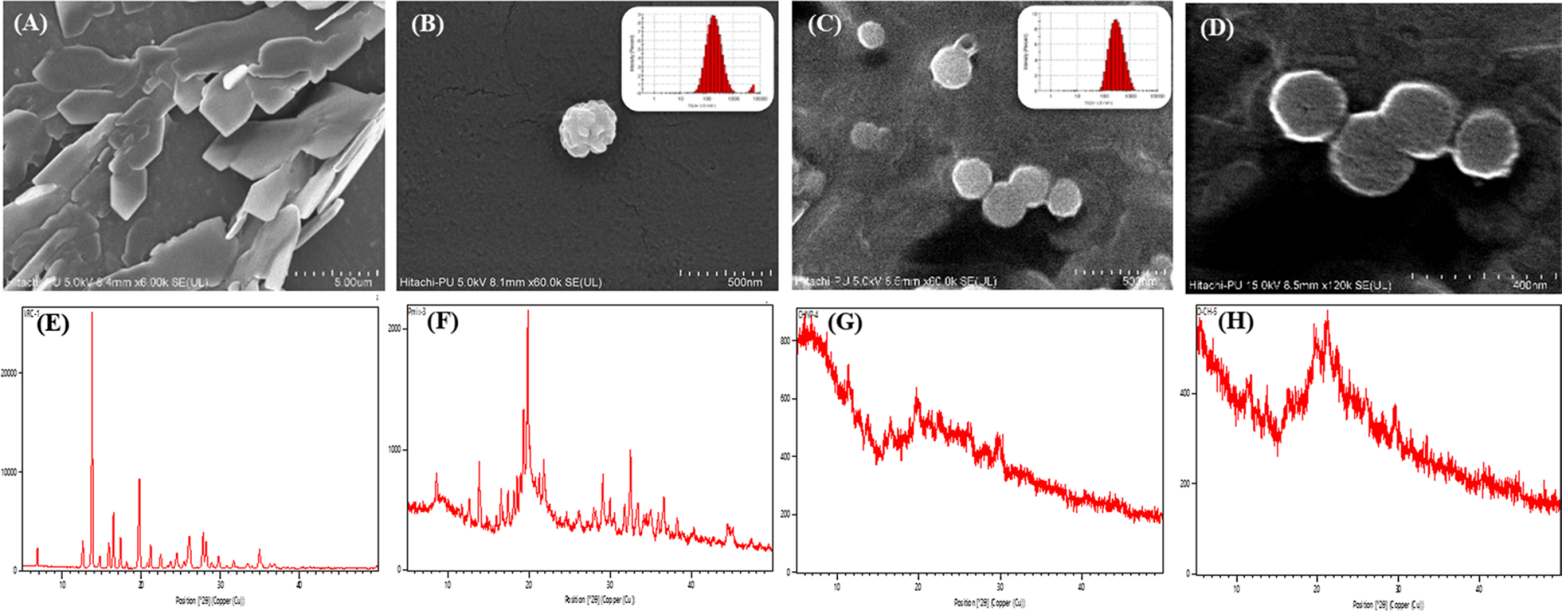
FESEM images of **a** Voriconazole at 6 K, **b** Chitosan nanoparticles at 60X (inset laser diffraction histogram), **c** Lipid-polymer hybrid nanoparticles at 60X (inset laser diffraction histogram) and **d** lipid-polymer hybrid nanoparticles at 120X; PXRD spectra of **e** Voriconazole, **f** physical mixture of drug and excipients, **g** Chitosan nanoparticles and **h** lipid-polymer hybrid nanoparticles

Varying the proportions of polymer, cross-linker, and surfactant contributed to the formation of either clear, opalescent or turbid solutions, with a mean PS ranging between 83.5 and 781.3 nm, ZP between 8.3 and 25.1 mV and EE between 26.3 and 48.2%. The desired region of CH NPs was obtained in the opalescent zone, where the polymer as well as cross-linker, was optimally used to form an ionic complex. Overall, the optimized CH NPs dispersion showed a PS of 174.00 ± 14.41 nm, PDI of 0.36 ± 0.05, ZP of 17.70 ± 0.42 mV, EE of 40.23 ± 4.76% (indirect method) and 35.03 ± 6.52% (direct method), and DL of 5.42 ± 1.01%. Coating the CH NPs with DPPC resulted in spherical shaped LPH NPs with a smooth surface, as is evident from their FESEM images (Fig. 1 a-d). This was in sheer contrast to the rough surface demonstrated by the corresponding images of CH NPs. Presence of lipidic layer on to the surface of LPH NPs was found to increase the PS and decrease the ZP (Additional file 1: Table S4) [55, 56]. Presence of lipid coat augmented the drug EE by 1.3-folds, ostensibly owing to the presence of the adsorbed drug on the surface of LPH NPs. The physicochemical characteristics of the optimized LPH NPs were found to be significantly different from the uncoated CH NPs with a mean size of 240.2 ± 12.10 nm (p < 0.005), ZP of 9.42 ± 0.33 mV (p < 0.001) and EE of 54.80 ± 5.19% (p < 0.05), as analysed using the indirect method. However, the difference in PDI (*i.e.,*0.28 ± 0.03), EE (*i.e.,* 45.46 ± 3.89) by direct method, DL (*i.e.,*6.28 ± 0.40%) was observed to be statistically insignificant (p > 0.05) for LPH NPs. A blend of mannitol (5%) and Trehalose (5%) was found to be more conducive for lyophilization of LPH NPs than either of them when used alone (Additional file 1: Table S5). The lyophilized LPH NPs exhibited a PS of 268.9 ± 15.16 nm, PDI of 0.30 ± 0.017, ZP of 8.25 ± 1.17 mV and EE of 52.86 ± 2.54%.

The FTIR spectrum (Additional file 1: Fig. S4) of voriconazole depicted peaks of C-N, C-F, and C–C stretching bands at 1099.80–1352.59, 1133.14–1408.38 and 1497.90 -1589.87 cm^−1^, respectively [57, 58]. The spectrum of CH showed peaks at 3399.90 cm^−1^ for O–H, 2856.30–3399.90 cm^−1^ for C-H stretching, and 1075.27 cm^−1^ for C-O stretching bands. Principal bands present in the DPPC spectrum included O–H stretching at 3038.68–3304.77 cm^−1^, C-H stretching at 2851.03–2954.40-28 cm^−1^, ester carbonyl groups at 1740.24 cm^−1^ and PO_2_^−^ groups at 1090.23–1231.45 cm^−1^ [59]. The corresponding spectrum of the physical mixture showed the sum of vibrational frequencies of all the individual components. The FTIR spectrum of LPH NPs primarily depicted the characteristic peaks due to the lipid and polymer. The peaks corresponding to drug molecule per se were not evident, thus rationally indicating the successful encapsulation of voriconazole. Figure 1(e–h) display the X-RD pattern of pure voriconazole, demonstrating sharp characteristic peaks at 13.8°, 16.5, and 19.8°, while such sharp peaks were almost absent in LPH NPs, indicating predominately the amorphous nature of drug embedded in the NPs [34].

### In vitro drug release studies

**Fig. 2:**
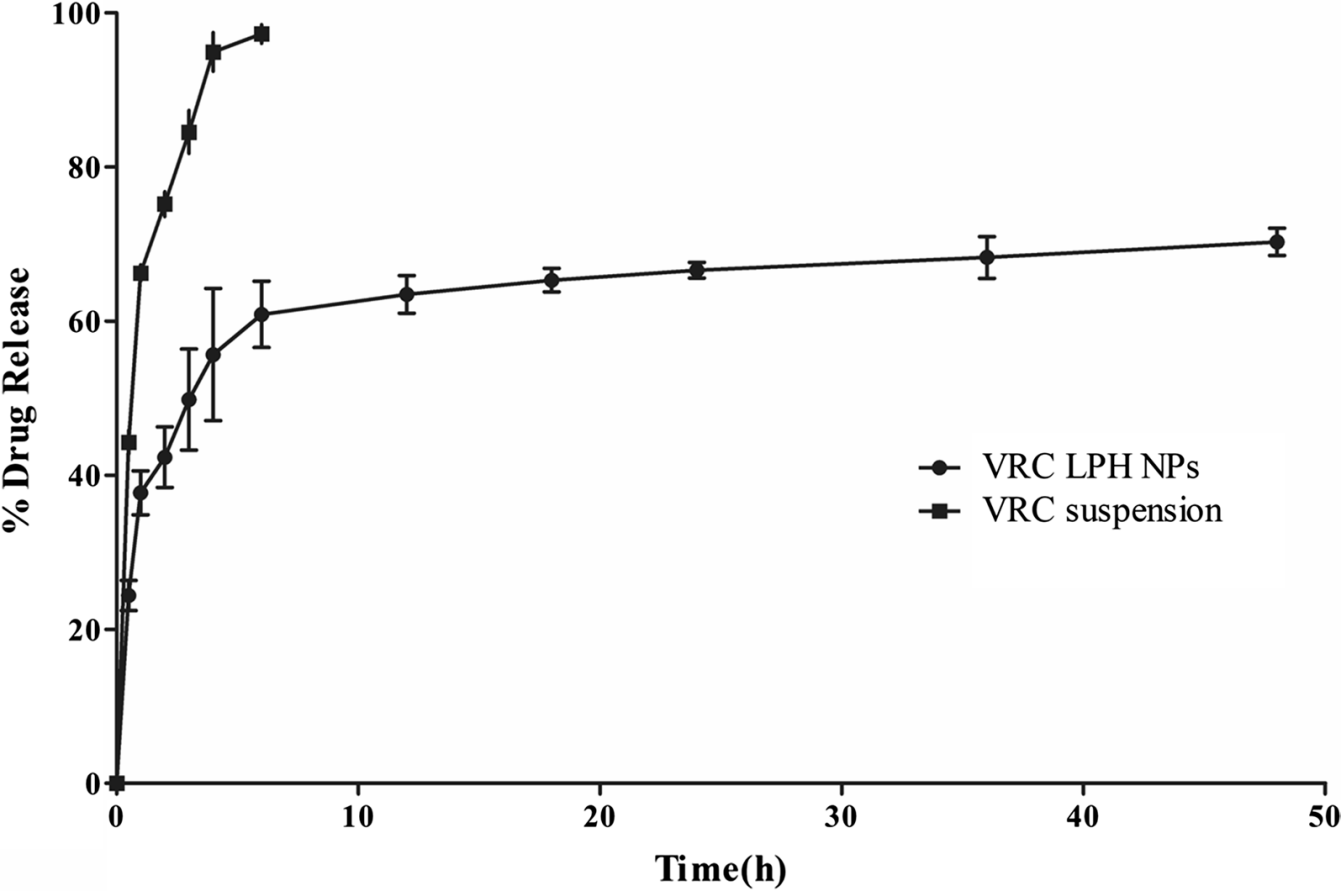
Cumulative drug release profile of voriconazole (VRC) and its optimised Lipid-polymer hybrid nanoparticles (LPH NPs) in PBS 7.4, for a period of 48 h. Data is expressed as mean ± SD (n = 3)

The drug release profile (Fig. 2) portrays that 94.94 ± 2.50% of voriconazole got released from voriconazole suspension in 4 h. The developed LPH NPs show extended drug release for 48 h (70.29 ± 1.77%), with an initial burst release for 2 h (42.33 ± 3.93%). The drug release profile was found to be best fitted with the Korsmeyer-Peppas model (R = 0.927), with the value of diffusional release exponent (n) being 0.202 (< 0.45), indicating the drug release to be primarily governed by Fickian mechanism (Additional file 1: Table S6).

### Microdroplet size analysis by spraytec

**Fig. 3:**
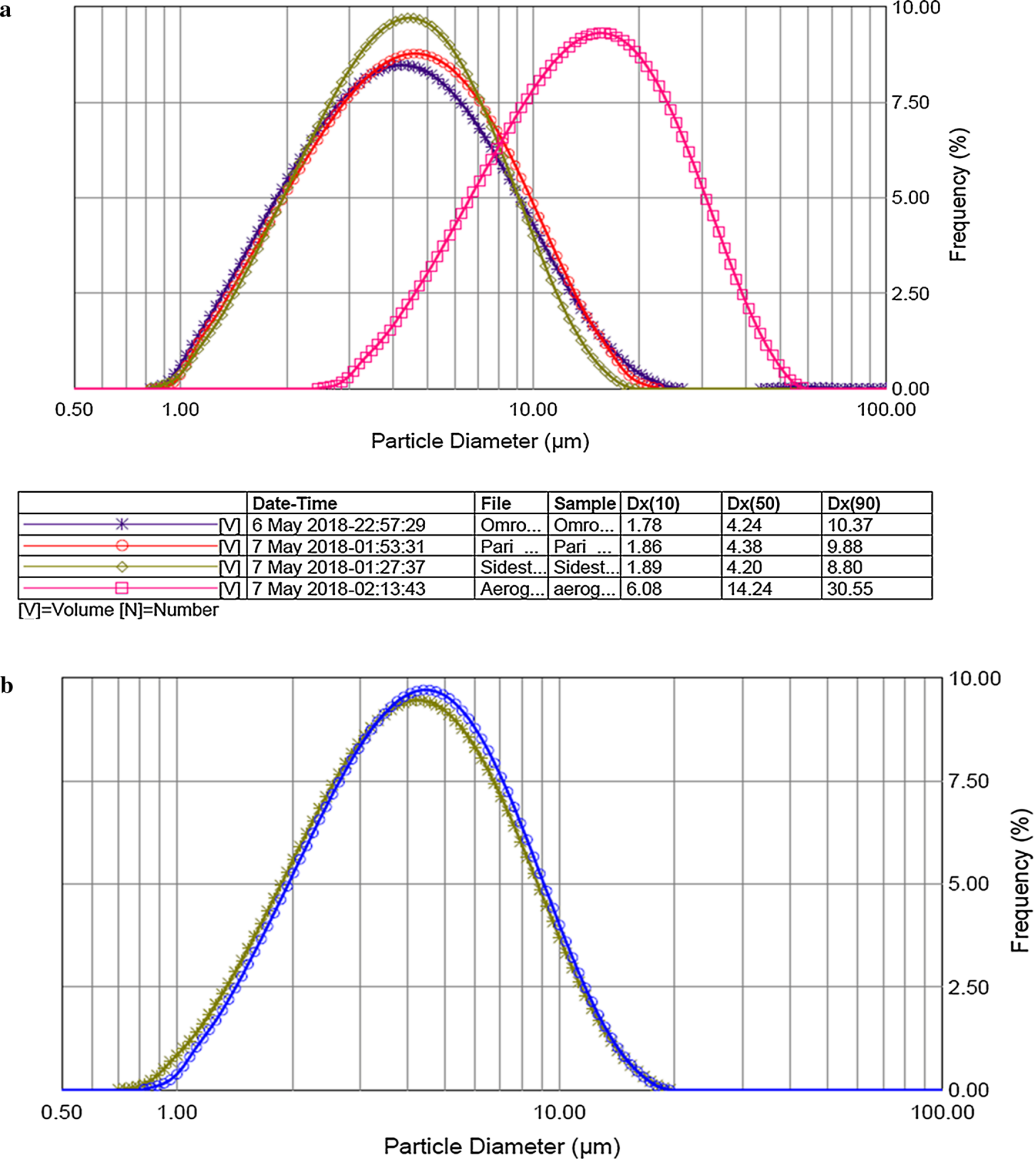

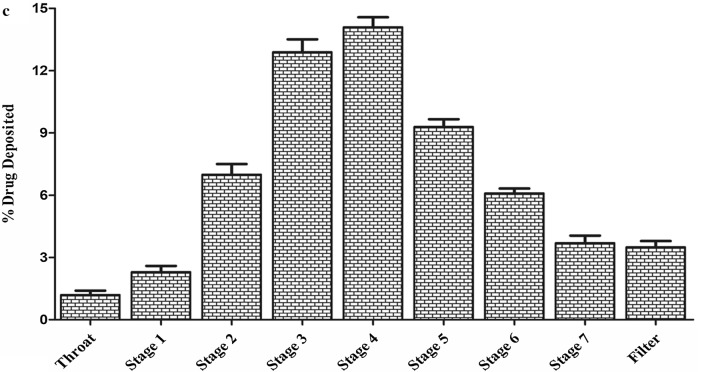
**a** Microdroplet characterization of lipid-polymer hybrid nanoparticles of voriconazole by Spraytec laser diffraction employing air-jet and mesh nebulizers at a distance of 3 cm from laser beam for 60 s, **b** microdroplet comparison of normal saline solution *versus* lipid-polymer hybrid nanoparticles using Sidestream nebulizer, and **c** next-generation impactor deposition pattern of lipid-polymer hybrid nanoparticles on various stages of impactor at a flow rate of 15 L. minute^−1^. Data is expressed as mean ± SD (n = 3)

Four nebulizers were investigated for their suitability to form appropriate inhalable microdroplets of LPH NPs dispersion. Other than Aeroneb Solo mesh nebulizer, which displayed Dx (50) of 14.24 µm, all other jet nebulizers exhibited Dx (50) of less than 5 µm (Fig. 3a). This could be ascribable to the blockage of the mesh of the Aeroneb Solo nebulizer by any free polymeric fibres that may be generated in the nanoparticulate dispersion during nebulization, resulting eventually in the generation of uneven droplet size(s). Other parameters like GSD, Span, percentage of the volume of particles less than 5 µm, etc., were also analysed for the spray pattern, as enumerated in Table 1. Based upon the results of Dx (50) µm, GSD, and  % volume of particles less than 5 µm, Sidestream nebulizer was found to be optimum for the nebulization of LPH NPs and was finally selected for evaluating the pulmonary deposition during the current studies. Further, the aerodynamic parameters obtained after the nebulization of normal saline solution employing Sidestream nebulizer were found to be insignificant (p > 0.05), when compared with LPH NPs nebulization, as depicted vividly in Fig. 3b.

**Table 1:** Comparison of performance characteristics of different nebulizers

Parameters	Aerogen Solo	Omron NE C803	Pari LC	Sidestream^$^
D_v_(10) µm	5.89 ± 0.88	1.82 ± 0.07	1.89 ± 0.06	1.90 ± 0.04
D_v_(50) µm	14.23 ± 1.21	4.37 ± 0.14	4.44 ± 0.19	4.20 ± 0.10
D_v_(90) µm	31.31 ± 0.60	10.17 ± 0.39	10.03 ± 0.63	8.76 ± 0.17
GSD	1.91 ± 0.15	1.89 ± 0.06	1.83 ± 0.02	1.73 ± 0.04
Span	1.77 ± 0.18	1.95 ± 0.04	1.83 ± 0.05	1.63 ± 0.01
%V < 5 µm	7.17 ± 4.22	58.92 ± 1.75	56.85 ± 2.60	61.08 ± 1.40
%V < 1-3 µm	1.55 ± 1.07	30.40 ± 1.73	28.60 ± 1.69	29.67 ± 1.24

Dv(10): Microdrolpets below which 10% spray lies, Dv(50): Microdrolpets below which 50% spray lies, Dv(90): Microdrolpets below which 90% spray lies, *GSD* Geometric standard deviation; $: nebulizer finally selected for performance evaluation. Data expressed as mean ± SD (n = 3)

### Particle deposition studies by NGI

The in vitro pulmonary deposition data of LPH NPs are presented as percentage of total drug deposited in the device, throat and various stages of NGI, with reference to the total mass recovered (Fig. 3c), revealing maximum drug deposition at Stage 4 (cut-off of 3.3 μm) and Stage 3 (cut-off of 5.4 μm) of the impactor, respectively. The MMAD and GSD were found to be 4.12 μm and 2.25 μm, respectively, with 86% of the dose emitted from the nebulizer, and an FPF as 55.5%.

### Effect of particle size on nebulization of LPH NPs

**Fig. 4:**
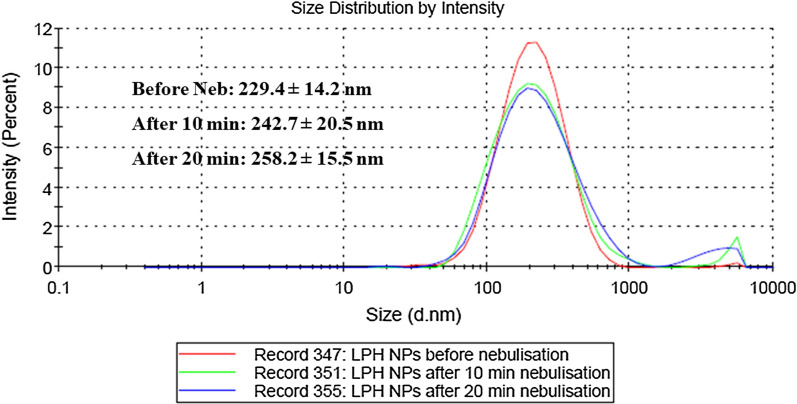
Effect of shear forces of Sidestream nebulizer on particle size of voriconazole lipid-polymer hybrid nanoparticles (LPH NPs) before and after nebulization of 20 min

The effect of shear forces of nebulizer on PS of LPH NPs is illustrated in Fig. 4. A marginal (258.2 ± 15.5 nm; p > 0.05) augmentation in the PS was observed following 20 min of nebulization (Table 2), thereby demonstrating the stability of the developed nanosystem during the process of nebulization.

**Table 2:** Effect of nebulization on particle size of LPH NPs

Parameter	Particle size	P-value
Before nebulization	229.4 ± 14.2	–
After 10 min of nebulization	242.7 ± 20.5	0.388 (ns)
After 20 min of nebulization	258.2 ± 15.5	0.076 (ns)

*ns* non-significant

### Surface activity experiments

**Fig. 5:**
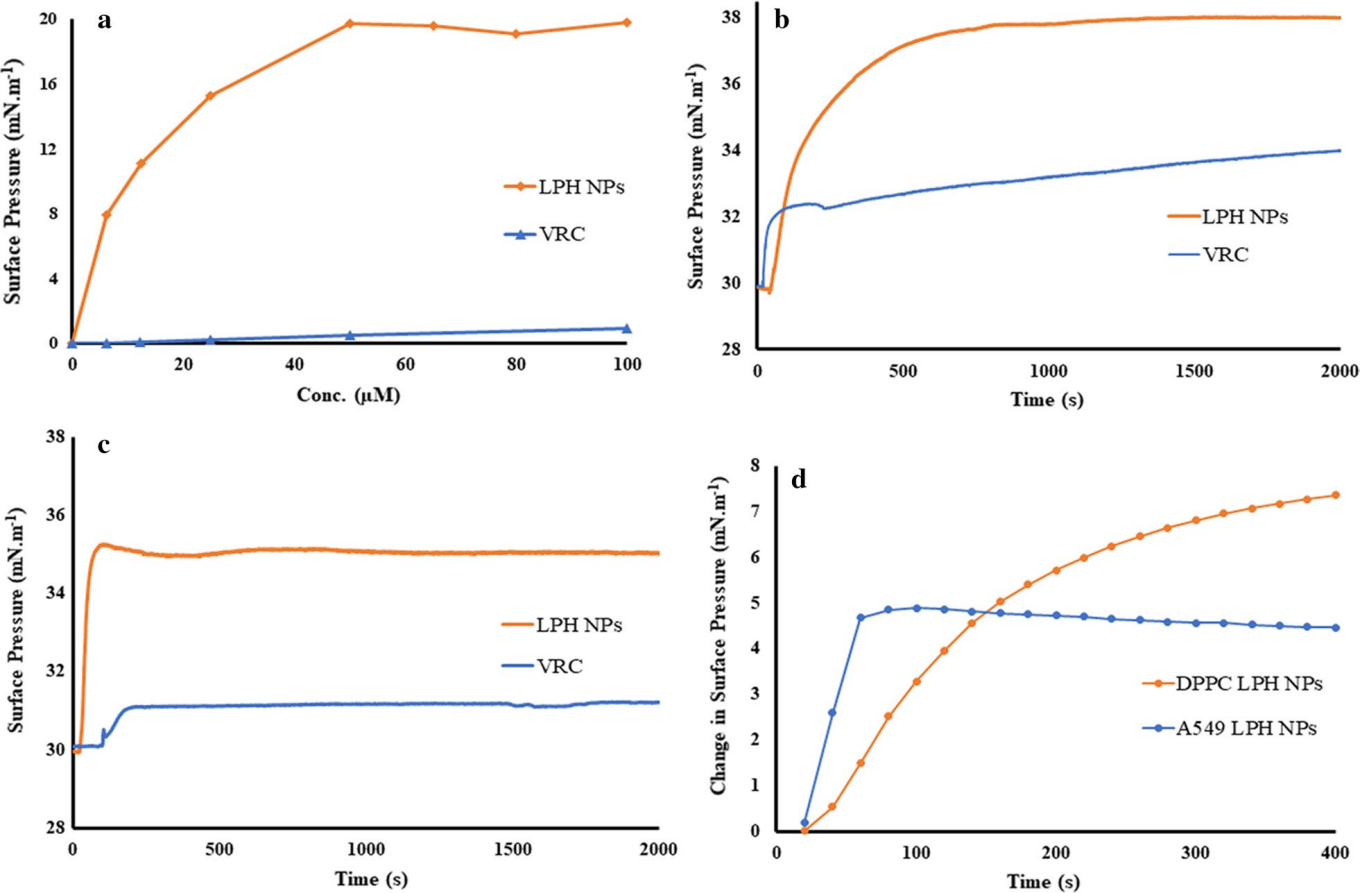
**a** Surface activity of voriconazole (VRC, blue) and lipid-polymer hybrid nanoparticles (LPH NPs, orange) at air/water subphase. Different compound concentrations (0–100 µM) were introduced into the subphase (10 mM PBS buffer, pH 7.4) of a Langmuir–Blodgett system, **b** DPPC monolayer interaction with VRC and LPH NPs for 2000s, **c** A549 cell lipid monolayer interaction with VRC and LPH NPs for 2000s and **d** change in surface pressure experienced with introduction of VRC and LPH NPs into lipid monolayers for initial 400 s

As portrayed in Fig. 5a, the surface pressure at air/water subphase tends to increase initially with increased drug concentration until saturation was achieved. For LPH NPs, the bulk concentration of 50 µM was observed to be the minimum requirement for saturating the air/water interphase, indicating that the voriconazole NPs were more surface-active than native voriconazole (p < 0.05). Furthermore, at the minimum bulk concentration, LPH NPs were observed to be highly surface-active displaying surface pressure increase of 19.75 ± 1.28 mN m^−1^, which is a characteristic of the membrane interactive compound [60].

#### Interactions of drug and nanoparticles with lipidic monolayers

Native voriconazole displayed minimal surface activity with a change in pressure (Δπ) of less than 1 mN m^−1^ (Fig. 5b), thereby indicating nominal interaction with the phospholipid headgroups. On the other hand, LPH NPs illustrated Δπ of more than 3 mN m^−1^, indicating expeditious ingress and intercalation of the NPs into the erstwhile hydrophobic acyl chain region of the monolayer [61]. This surface pressure increase followed hyperbolic kinetics and showed maximal values that ranged between 36.9 and 38.0 mN m^−1^, when studied for a time period of 2000s, consistent with membrane interactions of the acyl chain region [62].

Analogous to the aforesaid findings obtained with DPPC monolayers, the LPH NPs showed a rapid increase in surface pressure (Δπ of 4 to 5 mN m^1^) upon interaction with the A549 lipid monolayer, thus revealing their fusogenic properties (Fig. 5c). A lower level of interaction, however, was observed for such monolayers, wherein voriconazole induced a modest surface pressure alteration *i.e.,* 1 mN m^−1^. The initial interaction of LPH NPs with both the monolayers for a period of 400 s was different from each other. A slower increase in surface pressure was observed with DPPC monolayers, while a rapid rise followed by stagnant phase was observed with A549 lipid monolayers (Fig. 5d).

### Antifungal studies

The MIC values for LPH NPs were observed to be much lower in magnitude for the studied laboratory strains, while equal in magnitude to voriconazole in the clinical isolates of Aspergillus species, and lower in magnitude for Candida species (Table 3).

**Table 3:** Values of minimum inhibitory concentration (MIC) of voriconazole (VRC) and lipid polymer hybrid nanoparticles against different fungal strains

Type of strain	Fungal strain	Labortory I.D.	MIC (µg. mL^−1^)		
			VRC	Blank LPH NPs	Drug loaded LPH NPs
Laboratory strains	*Candida krusei*	CK 6258	0.23	14.84	0.11
	*Candida parapsilosis*	CP 22,019	0.05	14.84	0.03
	*Aspergillus flavus*	ATCC 204304	0.92	14.84	0.46
	*Aspergillus fumigatus*	ATCC 204305	0.92	14.84	0.23
Clinical isolates	*Aspergillus flavus*	R 4443	0.92	14.84	0.92
	*Aspergillus fumigatus*	R 4322	0.92	14.84	0.92
	*Aspergillus fumigatus*	R 4784	0.92	14.84	0.92

*LPH NPs* lipid polymer hybrid nanoparticles, *R* Respiratory clinical isolate

### In vitro cell lines study

#### Safety studies

**Fig. 6:**
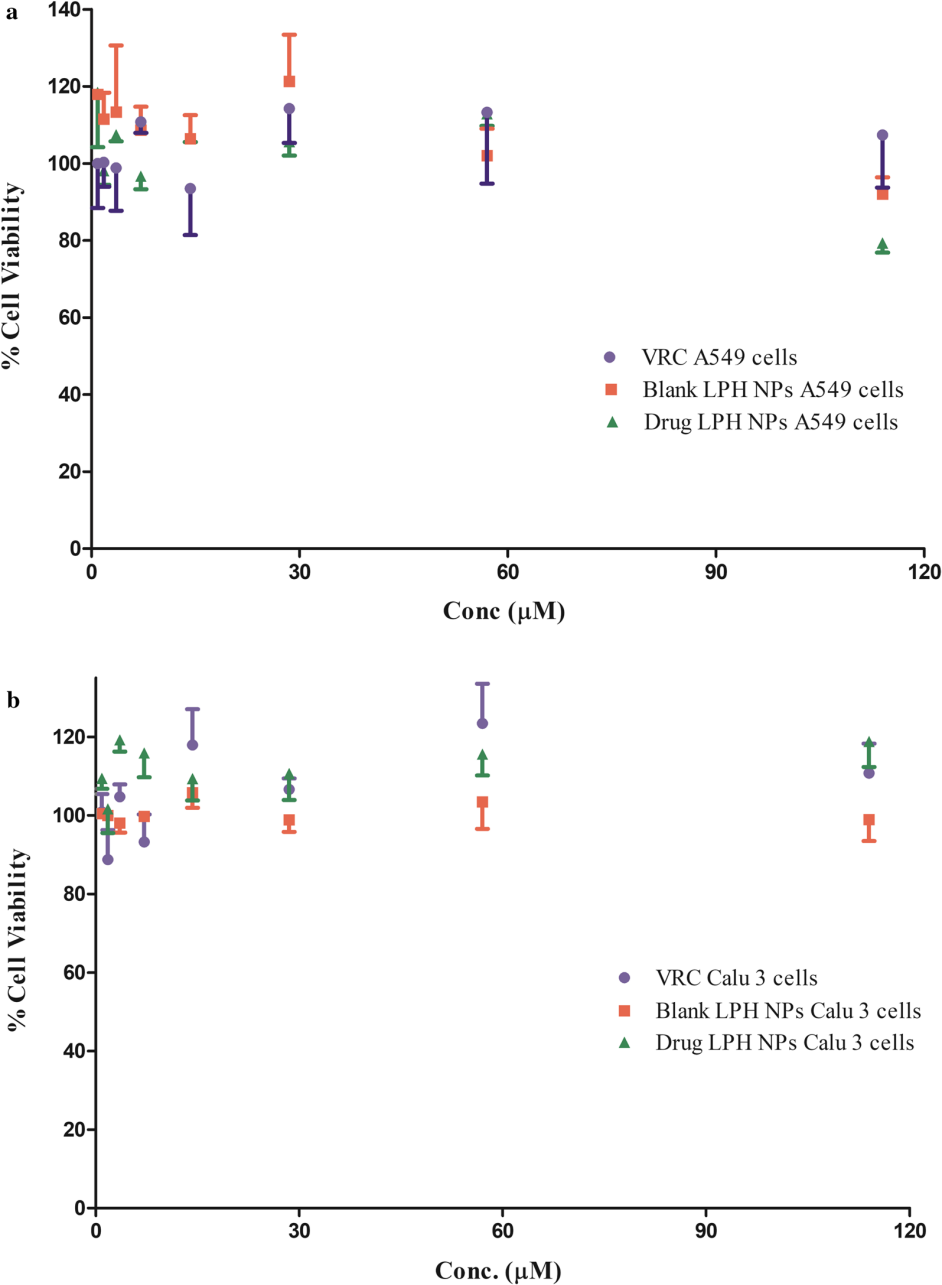
Cell viability assay of voriconazole (VRC) and lipid-polymer hybrid nanoparticles (LPH NPs) on **a** A549 cells and **b** Calu 3 cells for 48 h employing Presto Blue assay. Data is expressed as mean ± SD (n = 3)

Safety and compatibility of the formulation with the lung cells play a crucial role in developing a pulmonary drug delivery system, since the inhalation of any toxic substances may lead to depletion of lung surfactant and recruitment of phagocytic cells [9]. Both blank and drug-loaded LPH NPs did not compromise the cell viability, as more than 80% of cells were found to be viable as compared to control (p > 0.05), on being treated with the highest (40 µg mL^−1^) drug concentration (Fig. 6a, b).

#### Qualitative and quantitative uptake studies

**Fig. 7:**
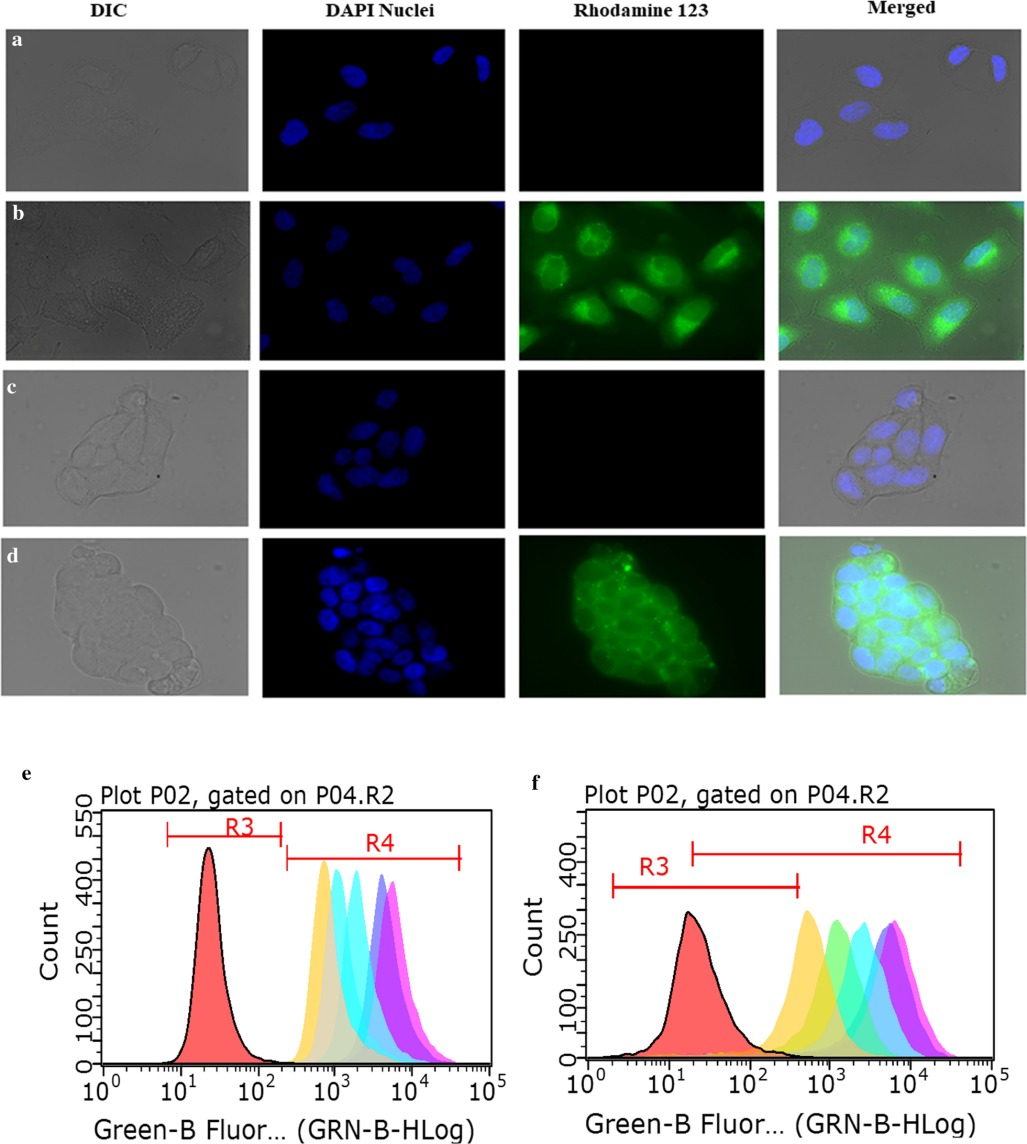

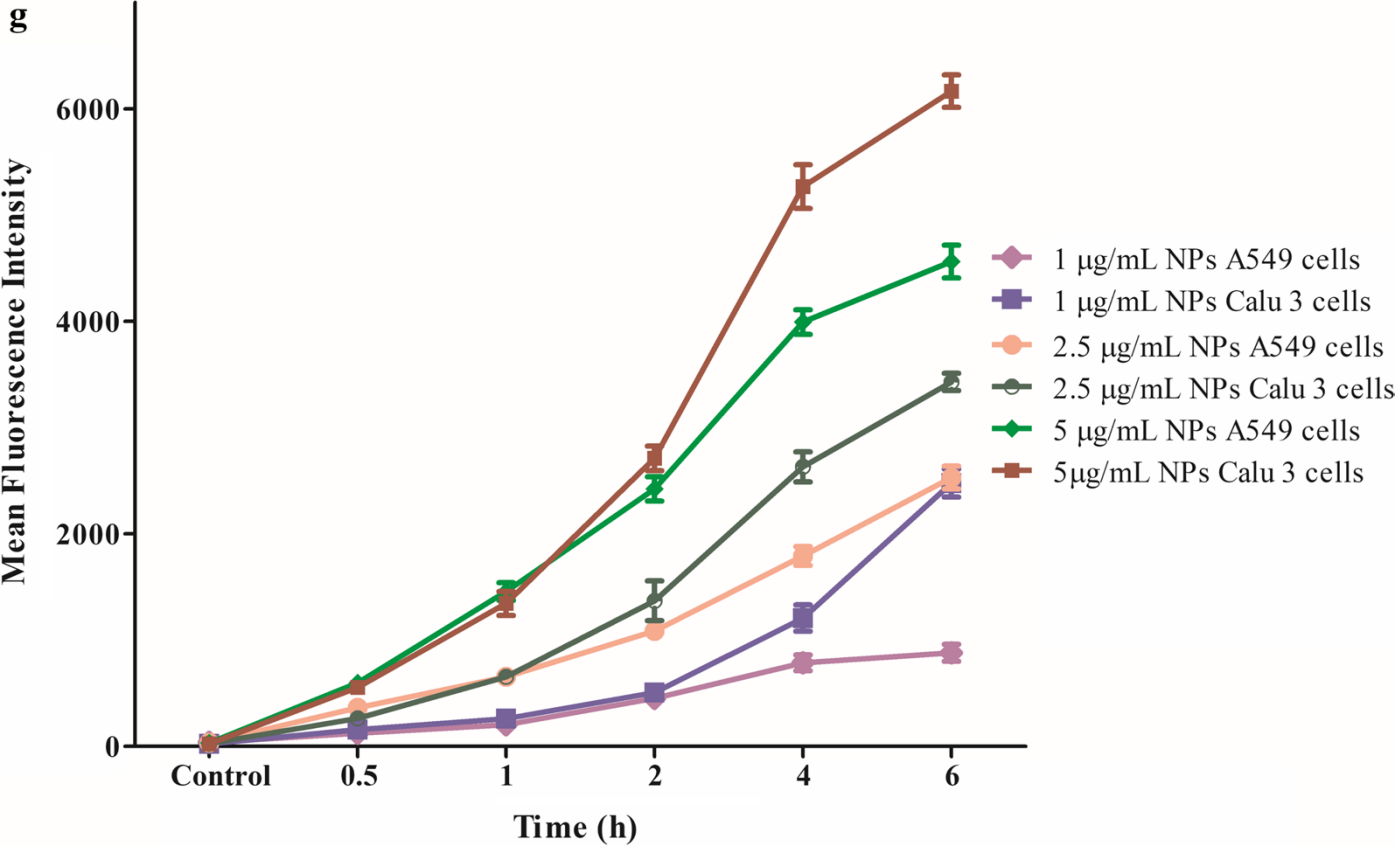
Fluorescence microscopic images of cellular uptake of Rhodamine 123 loaded lipid-polymer hybrid nanoparticles (10 µg.mL^−1^of R 123) depicted by Green fluorescence, Blue fluorescence-nuclei stained with DAPI, **a** A549 control cells, **b** A549 treated cells, **c** Calu 3 control cells, and **d** Calu 3 treated cells. Time-dependent uptake studies in **e** A549 3 cells and **f** Calu 3 cells. **g** Concentration-dependent uptake studies in A549 and Calu 3 cells. Data is expressed as mean ± SD (n = 3)

Fluorescent images of A549 and Calu 3 cells incubated for 4 h with Rh-LPH NPs reveal the presence of considerable green fluorescence inside the cells confirming the co-localization of Rh-LPH NPs in the airway cells, where blue colour represents the staining of the nucleus with DAPI, as portrayed in Fig. 7(a–d). It is evident that the cellular uptake of Rh-LPH NPs was both time-(Fig. 7e, f) and concentration-dependent (Fig. 7g). The internalization of NPs increases quite linearly with time, though they tend to plateau at higher levels beyond 4 h of treatment. Nearly 1.3-folds increase in the internalization was observed in Calu 3 cells vis-à-vis A549 cells at higher concentrations, for a period of 4 to 6 h.

#### Mechanistic pathway

**Fig. 8:**
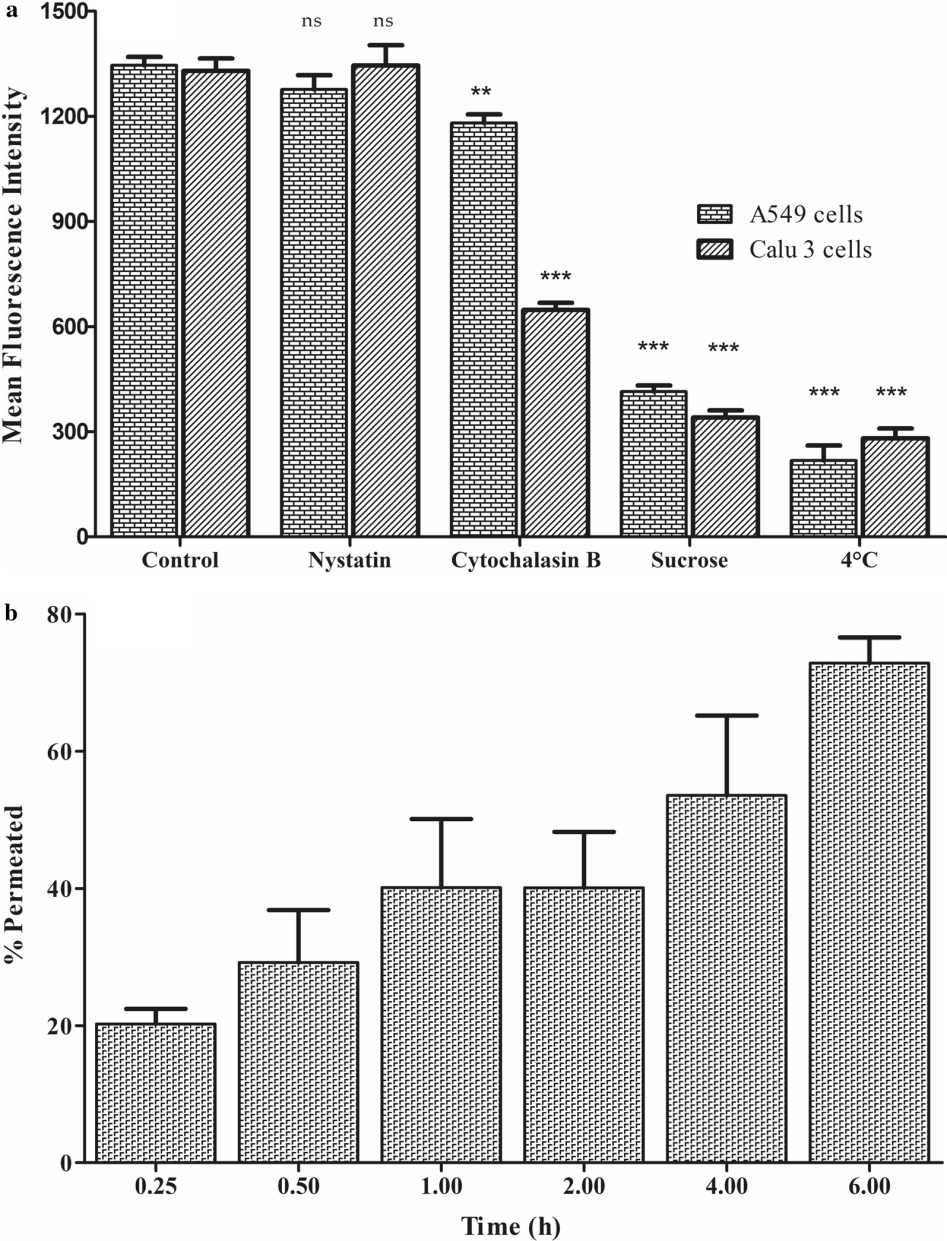
**a** Mechanistic pathways: Effect of endocytic inhibitor on cellular uptake of Rh123 labelled lipid-polymer hybrid nanoparticles in A549 and Calu 3 cells, **b** percent permeation of Rh 123 labelled lipid-polymer hybrid nanoparticles in Calu 3 monolayers for a period of 6 h. Data is expressed as mean ± SD (n = 3). *ns* nonsignificant, ^**^P < 0.001, ^***^P < 0.0001

Endocytosis enables cells to internalize nutrients, including macromolecules and NPs. Temperature-dependent uptake studies revealed that the energy-dependent active endocytic process was responsible for the uptake of Rh-LPH NPs in both the cell lines, as the internalization of NPs was reduced by five to sixfolds (p < 0.0001) w.r.t. control. NPs internalize in the cells by various mechanisms, and the preferred route of entry may depend on the properties of the carrier and the cell type [63]. Internalization of LPH NPs in A549 cells was primarily attributed to the clathrin-mediated pathway (*i.e.,* sucrose inhibition), as the uptake of NPs was reduced by 3.2-folds w.r.t. control (p < 0.0001), and significant effect was also observed with cytochalasin B (p < 0.001), a macropinocytosis pathway-specific inhibitor. In Calu-3 cells, however, two transport pathways were majorly involved, *i.e.,* clathrin-mediated and macropinocytosis as internalization was reduced by 3.9-and 2.1-folds (p < 0.0001 each), respectively in the presence of sucrose and cytochalasin B, the specific inhibitors for each of these pathways. Nevertheless, the insignificant effect was observed on internalization (p > 0.05) with nystatin on both the cell lines, thus ruling out the involvement of caveolae-dependent pathway for uptake (Fig. 8a and Additional file 1: Fig. S5).

#### Permeability studies

Reduction in TEER of Calu 3 monolayer, after exposure to NPs, w.r.t control (Additional file 1: Fig. S6) could be attributed to the slight opening of the tight junctions connoting paracellular transport, though the resistance remained well above the 1000 ohm cm^−2^ verifying that the integrity of the monolayer was intact [64]. The integrity of the monolayer was further confirmed by no evident leakage of Evans blue when challenged at the end of treatment. Percent permeation of LPH NPs, as illustrated in Fig. 8b, unequivocally construes the potential of the formulation to transport across Calu-3 cell layers, in consonance with our cellular uptake studies with the Papp of Rh-LPH NPs as 5.79 ± 0.86 × 10^−6^ cm s^−1^.

### Plasma and lung pharmacokinetic studies

**Fig. 9:**
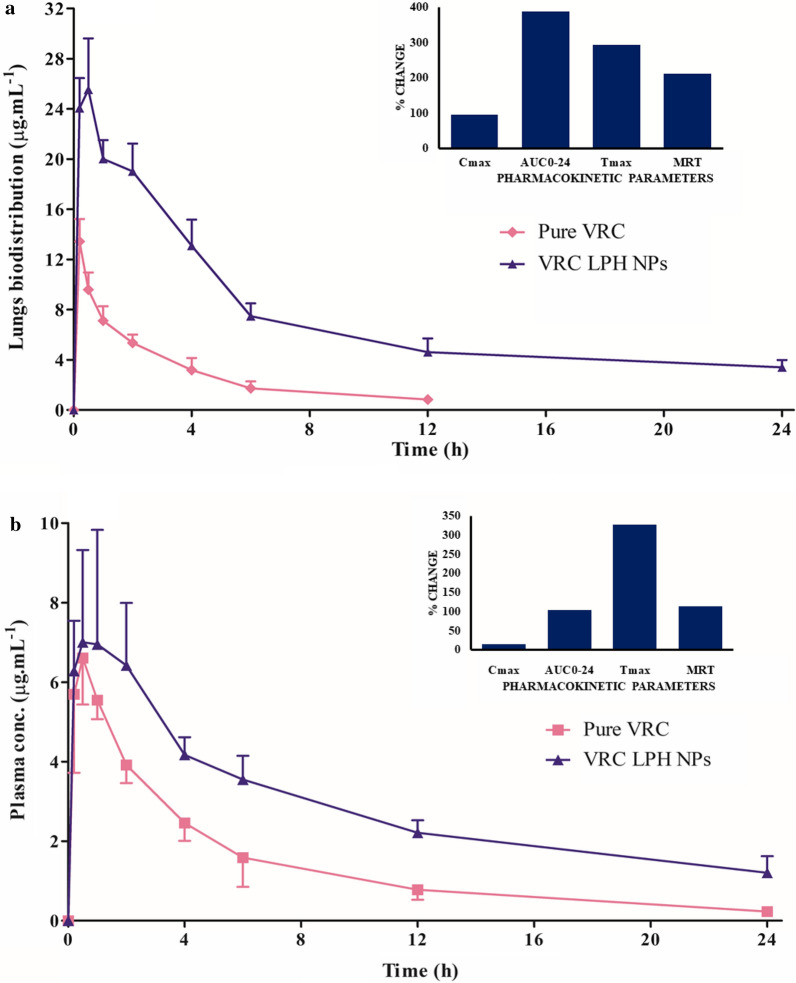
Pharmacokinetic profiles of voriconazole (VRC) and lipid-polymer hybrid nanoparticles (LPH NPs) at different time-points in (**a**) Lungs and (**b**) Plasma, following nebulization for 20 min to Balb/c mice through inhouse nose-only inhalation chamber. Data expressed in mean ± SD, n = 3 mice/group. The corresponding inset depicts percent change in different pharmacokinetic parameters

The mean lung concentration–time profiles of voriconazole solution (Fig. 9a) show a rapid decline of drug levels following nebulization vis-à-vis that obtained with LPH NPs, thus leading to a distinct change in varied pharmacokinetic parameters (Table 4). As much as 4.9-folds (p < 0.0005), 5.7-folds (p < 0.0001), 3.1-folds (p < 0.005), 3.9-folds (p < 0.01), and 2.0-folds (p < 0.05) augmentation in the values of AUC_0-24_, AUC_0-**∞**_, MRT, T_max_ and C_max_ was observed, respectively, with LPH NPs w.r.t. pure voriconazole.

**Table 4:** Pharmacokinetic parameters following nebulization of voriconazole (VRC) and its lipid-polymer hybrid nanoparticles (LPH NPs)

Parameter(s)	Lung pharmacokinetics		Plasma pharmacokinetics	
	VRC solution	VRC LPH NPs	VRC solution	VRC LPH NPs
C_max (_μg mL^−1^)	13.43 ± 3.10	26.29 ± 5.78	6.81 ± 1.11	7.79 ± 2.07
T_max_(h)	0.17 ± 0.00	0.67 ± 0.29	0.39 ± 0.19	1.67 ± 0.38
AUC_0-24 (_μg mL^−1^*h)	36.38 ± 5.40	177.75 ± 23.40	33.89 ± 5.05	69.01 ± 10.25
AUC_0-∞ (_μg mL^−1^*h)	41.77 ± 6.75	237.51 ± 22.98	35.94 ± 6.11	89.16 ± 16.71
MRT (h)	5.27 ± 1.31	16.46 ± 2.87	7.13 ± 1.03	15.26 ± 3.44

Data expressed as mean ± SD (n = 3)

On the other hand, plasma level time profile of voriconazole following nebulization (Fig. 9b), the mean values of T_max_ were observed to be 1.67 h for LPH NPs vis-à-vis 0.39 h (4.3-folds, p < 0.01) for the marketed formulation. The AUC_0-24_ (2.0-folds), AUC_0-**∞**_ (2.4-folds), MRT (2.1-folds) were also found to be significantly higher (p < 0.05 each) for the LPH NPs w.r.t. the marketed brand. The magnitude of C_max_, however, was not found to be significantly different (1.1-folds, p > 0.05).

### In vivo safety studies

**Fig. 10:**
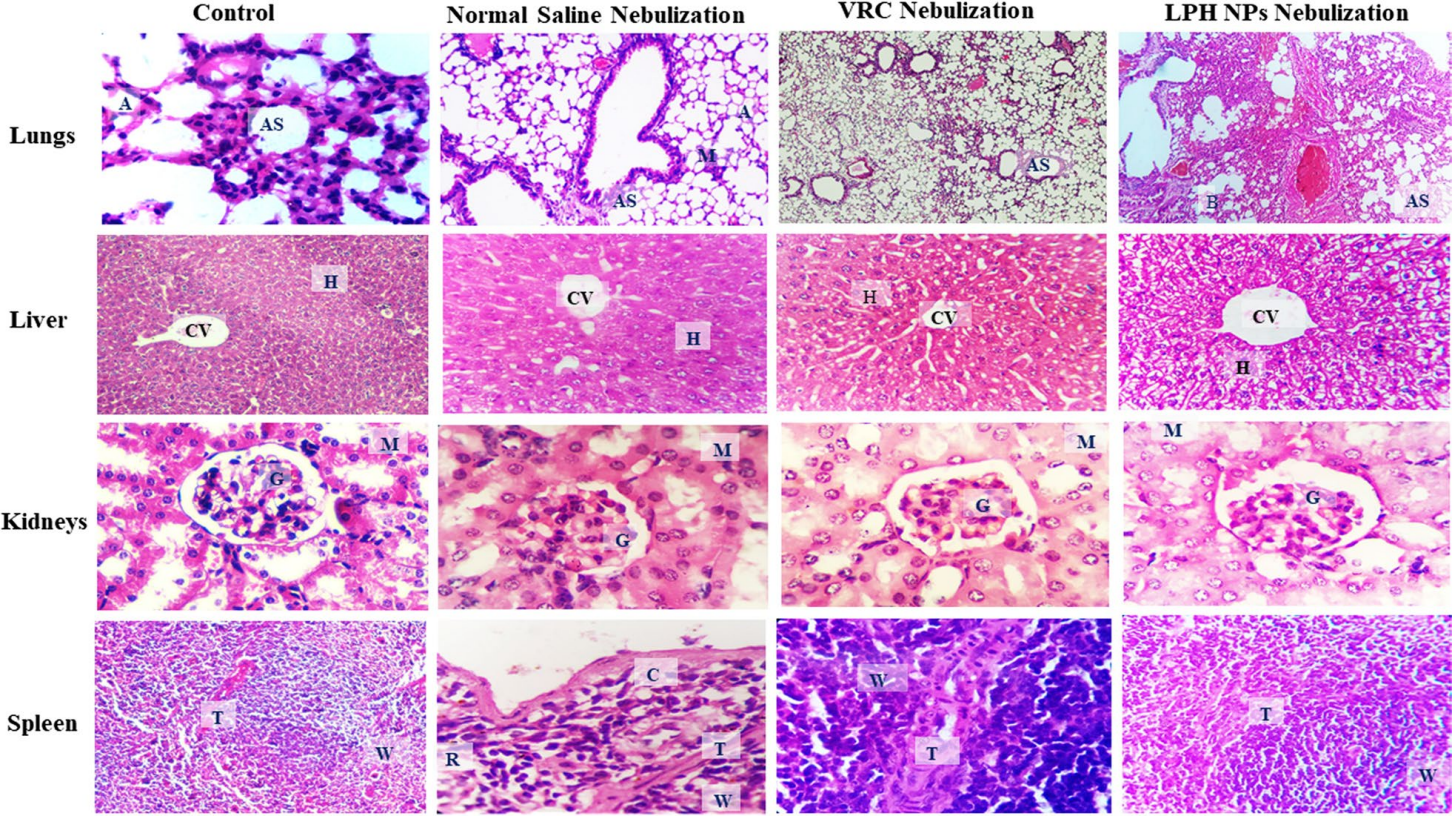
Photomicrographs showing histopathological changes in various organs of Balb/c mice after receiving twice daily nebulisation of **a** control, no treatment, **b** normal saline, **c** VRC and **d** LPH NP dispersion for a period of 21 days. *AS* alveolar sac, *CV* central vein, *H* hepatic vein, *G* glomerulus, *M* medullary vein, *T* trabecula, *W* white pulp, *R* red pulp, *C* capsule

Histopathological descriptions of sections of various Balb/c mice tissue specimens, viz. lungs, liver, spleen, and kidney, upon repeated nebulization of LPH NPs twice daily for a period of 21 days, showed only insignificant changes vis-à-vis the corresponding naïve group (Fig. 10). Mild distortion, however, was observed in the mice lungs treated with voriconazole and LPH NPs of voriconazole. However, no significant pathological changes, *i.e.,* ulceration of the airways or oedema, was observed, in concurrence with the results reported by Tolman et al. [50]. The liver of the treated groups exhibited no signs of distortion as parenchymal cells, and the central vein was distinct. Similarly, the architecture of spleen in all the groups was quite normal, with an intact trabecula and capsule. No signs, whatsoever, of the medullary cyst or nephropathy, were noticeable in the test population, though a mild increase in the space of glomerulus capsule was observed for LPH NPs vis-à-vis control.

### Stability studies

Table 5 demonstrates the stability data of LPH NPs at different storage conditions. LPH NPs were found to be stable at 5 °C for a period 90 days vis-à-vis at higher temperatures. No notable changes were observed at 25 °C for 30 days, while significant increase in PS and reduction in EE were observed after 90 days.

**Table 5:** Stability study data for freeze-dried hybrid nanoparticles of voriconazole

Parameters	Zero day	After 30 days			After 90 days		
		5 ± 3 °C	25 ± 2 °C/60 ± 5% RH	40 ± 2 °C/75 ± 5% RH	5 ± 3 °C	25 ± 2 °C/60 ± 5% RH	40 ± 2 °C/75 ± 5% RH
PS (nm)	270.2 ± 17.3	273.2 ± 21.14	278.6 ± 33.02	405.9 ± 31.75	282.8 ± 30.11	478.3 ± 40.85	531.5 ± 37.89
PDI	0.312 ± 0.012	0.373 ± 0.018	0.389 ± 0.047	0.431 ± 0.194	0.349 ± 0.024	0.580 ± 0.104	0.871 ± 0.117
EE (%)	52.25 ± 2.72	51.72 ± 2.81	49.34 ± 3.01	43.92 ± 3.57	50.43 ± 3.91	44.84 ± 3.48	36.67 ± 4.59

*PS* Particle size, *PDI* polydispersity index, *EE* Entrapment efficiency

## Discussion

With the growing incidences of aspergillus lung infections, the ardent need of the hour today is to make efficacious therapeutic strategies available for its prevention and eradication, demonstrating efficient targeting to the infected cells vis-à-vis invasive parenteral drug delivery approaches. Development of safe, effective, biocompatible localized pulmonary therapy is, accordingly, called for to address this impending vital issue. Concerted as well as collaborative endeavours have, therefore, been undertaken in the current studies to develop LPH NPs of a standard antifungal drug, voriconazole, in order to attain its efficient localized therapy.

The standard protocol of electrostatic interaction was opted to prepare LPH NPs. The solubility of CH in 1% acetic acid solution was obtained ostensibly due to protonation of an amine group of CH into ammonium ions, as acetic acid in solution gets converted to acetate ions (CH3COO^−^) and (H^+^) ions [65]. The positive charge on the CH NPs was predicted to attract negatively charged acetate ions that shield the cationic centres from interacting with the negatively charged lipids. The ammonium (NH_4_^+^) groups present in the zwitterionic DPPC may divert the negatively charged acetate ions from the acetic acid solution, thus enabling more frequent interactions between the anionic phosphate groups of DPPC and the cationic surface of CH NPs. Moreover, the high-energy input by sonication seemed to facilitate the lipid organization around the CH NPs surface. Presence of DPPC coating was confirmed by an increment in the PS and diminution in the ZP [55, 65]. Further, the FESEM images distinctly revealed perceptible changes in the morphology between CH NPs and LPH NPs, rationally assignable to the contribution of the lipid layer in providing a smooth surface and spherical shape to the latter compared to the relatively rough surface and irregular shape of the former. Difference in the values of EE of LPH NPs between the indirect and direct methods can ostensibly be ascribed to adsorption of some amount of drug onto the surface of the NPs. The presence of any sharp crystalline peak(s) of voriconazole was almost absent in the XRD patterns of LPH NPs, which principally ratify the amorphous character of the prepared NPs.

PBS was employed as the dissolution medium owing to its simple composition, better buffering effect and aptness for demonstrating sustained release characteristics or pH-dependent profile [66]. Further, the pH of PBS was fixed at 7.4 to reflect the pH of lung lining fluid [67]. LPH NPs portrayed a typical biphasic drug release profile in PBS 7.4, with an early short period burst release, followed by a relatively long controlled release phase for a period of 48 h. This initial burst release could be attributed to a sizeable amount of the drug adsorbed onto the surface of LPH NPs [68]. The subsequent controlled release could be ascribed to the contribution of CH in these lipid-polymer hybrid nanoconstructs.

The primary goal of drug delivery via inhalation is to achieve the desired drug levels in the lungs. Globule size is one of the major factors known to influence particle deposition in the lungs. NPs, owing to their smaller size, have demonstrated low deposition efficiency. Therefore, LPH NPs were nebulized employing a jet nebulizer for the generation of NPs-embedded into microdroplets with MMAD and VMD ranging between 1 and 5 µm, as particles > 5 µm are documented to get deposited in the large conducting airways as well as in oropharyngeal region [69]. MMAD (4.12 µm) of LPH NPs dispersion as determined using NGI, delineates that the aerosol possesses the ability to reach smaller airways, alveoli, and deposits primarily through the mechanism of sedimentation and diffusion in the airways. Furthermore, VMD (4.20 µm) calculated from Spraytec (p > 0.05) corroborates well with the results obtained using NGI. The variability in dispersity of aerosol droplets was predicted by GSD, where GSD value of 1 tends to construe a monodisperse aerosol, while it indicates a heterodisperse aerosol for a GSD value > 1.2 [69]. The results obtained with the current studies showed hetero dispersity of the generated aerosol cloud in concurrence with previous findings [69, 70]. The size and integrity of NPs, nevertheless, were quite well-maintained within the nebulized droplets, without any perceptible aggregation [71].

Langmuir–Blodgett air–liquid interphase monolayer models are widely accepted for investigating the interaction of cellular lipids with therapeutic moieties, as they mimic the naturally occurring membrane interfaces [45, 62]. The present studies, in this regard, demonstrated the distinct surface-active potential of LPH NPs vis-à-vis pure drug attributed to the DPPC coating on the CH NPs. Indeed, the membrane interactive potential of LPH NPs could help explain the kinetics of insertion into monolayers of DPPC and lung surfactant mimicking endogenous membrane comprising of A549 lipid extract (Fig. 5). The insertion of LPH NPs into the DPPC monolayers took 500 s to achieve maximal surface pressure (Fig. 5b), whereas only 50 s (Fig. 5c) for intercalation into A549 lipid monolayers. These data clearly suggest that LPH NPs not only enhances the interaction into DPPC and A549 monolayers but also facilitates rate of insertion into these membranes, indicating that the NPs has enhanced safety and stability, which can be accredited to the DPPC coating on the NPs.

In vitro compatibility studies revealed the safety of the LPH NPs in bronchoalveolar epithelial cells at the highest concentration tested. The qualitative data illustrated the co-localization of the developed nanosystems and exhibited their accumulation in the cytoplasm of lung epithelial cells, while the quantitative analysis revealed that the internalization of LPH NPs increased with time and dose. Interestingly, this augmentation appeared to be steeper at higher concentration of NPs with Calu 3 cells, displaying higher internalization than observed with A549 cells. As the positively charged NPs are known to internalize by clathrin-mediated endocytosis (CME), this pathway was considered primarily responsible for the internalization of the positively charged LPH NPs in the current studies, for A549 as well as Calu-3 cells. Electrostatic interactions seemed to be primarily involved in the receptor-independent CME [47, 72, 73]. As NPs usually employ multiple pathways to internalize, macropinocytosis, postulated to be involved in the uptake of both positively and negatively charged NPs, was also construed as one of the likely internalisation pathways of LPH NPs in Calu 3 cells [74]. For studying NPs translocation, Calu-3 cells are the most relevant respiratory cell culture model, because of their ability to form tight monolayers vis-à-vis A549 cells [75]. In the current study, the high magnitude of Papp, *i.e.,* > 5 × 10^−6^ cm s^−1^ for LPH NPs across Calu 3 monolayers, connotes the distinctive potential of the nanosystems to open tight junctions and transport across a monolayer of the cells [76].

The in vitro antimicrobial data on fungal isolates unravelled the potential of developed nanosystems to target the most prominent strain of fungal lung infections viz., *Aspergillus fumigates* responsible for causing more than 60% of the lung infections [8], followed by other Aspergillus and Candida species.

Currently, no inhalational formulation of voriconazole being commercially available, intravenous injection was nebulized to compare the lung deposition and plasma concentration profile of LPH NPs in mice. A previous report on the inhaled voriconazole injection has shown improved survival in the murine model of *Aspergillus fumigatus* infection [77]. Loading voriconazole into LPH NPs evidently revealed high lung retention vis-à-vis the marketed voriconazole solution. A notable improvement in the values of C_max_ and T_max_, reveal significant enhancement in the intensity and duration of voriconazole exposure to lungs on nebulisation of LPH NPs. This could be hypothesized to the ability of LHP NPs to circumvent all the three pulmonary barriers, *i.e.,* physical, biochemical and cellular [23]. In the current studies, as the NPs exhibited size less than 0.5 μm, they were apparently capable of overcoming the physical barrier and settle in the lungs by the mechanism of Brownian diffusion [78]. Further, modification of CH NPs with pulmonary surfactant lipid, viz, DPPC, could be postulated to impede the adhesion of NPs with mucus biochemical barrier and escape deposition in the upper airways [79]. Once the NPs reach the alveolar spaces, the DPPC coating facilitates their interfacial spreading at the air lung interface [80]. This is followed by intercalation into the PSM where they are exposed to highly dynamic environment and resist lung clearance owing to their surface-active characteristics [81, 82]. Integration of NPs with PSM promotes their interaction with alveolar epithelial cells and enhances their internalization through cellular pathways, thereby improving the AUC values and residence time of voriconazole in the lungs [23]. Native voriconazole with marginal surface activity is prone to removal during the constant compression and expansion of PSM during breathing, which could be the plausible reason for its fast clearance with no detectable levels of the drug in the lung tissue after 12 h [83].

The enhanced values of T_max_ in the plasma are ascribable to the slower diffusion of LPH NPs from the lungs, thereby allowing enough time for the cellular uptake of these nano-contoured systems within the lungs. The enhanced AUC values in plasma could plausibly be assigned to the presence of large alveolar surface area and thin physiological membrane in the lungs, which tend to promote drug diffusion into the systemic circulation [84, 85]. The results, therefore, signify the immense potential of LPH NPs for the management of invasive fungal disorders.

Safety of the developed formulation is vital for its potential clinical application(s). The toxicity studies revealed insignificant changes with no obvious organ damage either to the lungs or to the other vital organs, including heart, kidneys, liver, and spleen, following repeated-dose administration for 21 days. This corroborates the safety and biocompatibility of the LPH NPs upon nebulization to mice, which could be judiciously attributed to the usage of lung endogenous phospholipid, which has shown high potential to mimic the normal lung surfactant.

Lyophilization was shown to enhance the stability of colloidal NPs. LPH NPs, lyophilized with 5% mannitol and 5% trehalose, get easily dispersed in water, upon reconstitution. The presence of trehalose as cryoprotectant is known to enhance the stability of the NPs during freeze drying [86]. This could be postulated due to the physical isolation of adjacent NPs in the presence of glassy cryoprotectant to reduce the destabilising stress caused during the freezing process [87]. Significant changes (p < 0.05) in particle size were noted in LPH NPs stored at 40 °C, which could be rationally hypothesized to the aggregation of LPH NPs at higher temperatures [88]. Reduction in EE at 40 °C, could be attributed to the leakage of VRC from the NPs exposed to high temperature for prolonged period of time [86]. Accordingly, refrigeration conditions of 5 ± 3 °C would be conducive for storing LPH NPs for a 90-day period.

## Conclusions

Inhalation of antifungals has been emerging as an essential and effective therapy for the therapeutic management of fungal lung infections. Voriconazole-a first-line triazole usually gets rapidly cleared into the systemic circulation with low lung retention profiles, following nebulization. The current investigation, therefore, presents a novel inhalational delivery system comprising of lung-specific phospholipid coated CH NPs to augment the deposition and retention of voriconazole in the lungs. Various characterization studies revealed robust nanostructured characteristics of the prepared system with a modulated drug release profile and surface-active properties. In vitro pulmonary deposition, studies ratify the distinct potential of the nebulized NPs to target the lungs, primarily through the mechanisms of sedimentation and diffusion. Biophysical interaction of LPH NPs with lipid-monolayers confirm their surface-active potential and intercalation into the pulmonary surfactant barrier at the air-lung interface. In vitro cellular viability and in vivo histopathology studies construed safety potential and biocompatibility of the prepared lipid polymer nanocarrier system. The pharmacokinetic studies delineated enhanced lung retention with improved biopharmaceutical attributes. In vitro efficacy in laboratory and clinical isolates of Aspergillus species prove their potential in treatment and prophylaxis of PAP as secondary infection in many respiratory conditions. Further research will be focused on evaluating the dose-dependent pharmacodynamic response of LPH NPs in the validated animal models. The knowledge and know-how gathered from the current research work would enable future researchers working in the realm of inhalational drug delivery to develop the next-generation smart aerosol systems with demonstrable evidence to improve lung targeting.

## Supplementary information

**Additional file 1**: Tables and figures.

## Data Availability

The data generated and analysed during the current study is provided in the manuscript and is available from the corresponding authors on reasonable request.

## Abbreviation

AmB: Amphotericin B
AUC: Area under the curve
BBD: Box-Behnken design
CH: Chitosan
Cmax: Maximum concentration
CME: Clathrin-mediated endocytosis
CMAs: Critical method attributes
CQAs: Critical quality attributes
DLS: Dynamic laser scattering
DPPC: Dipalmitoylphosphatidylcholine
EE: Entrapment efficiency
FESEM: Field Emission Scanning Electron Microscope
FTIR: Fourier Transformed Infra-Red
HPLC: High performance liquid chromatography
LPH: Lipid-polymer hybrid
MMAD: Mass median aerodynamic diameter
MRT: Mean residence time
NGI: Next-generation impactor
NPs: Nanoparticles
PAP: Pulmonary aspergillosis
Papp: Apparent permeability
PDI: Polydispersity index
PS: Particle size
PSM: Pulmonary surfactant monolayer
QbD: Quality by design
Rh-123: Rhodamine 123
T80: Tween 80
TEER: Transepithelial electrical resistance
Tmax: Time to reach maximum concentration
TPP: Sodium tripolyphosphate
VMD: Volume mean diameter
X-RD: X-ray diffractometer
ZP: Zeta potential
