# Supplementary material for: Nebulised surface-active hybrid nanoparticles of voriconazole for pulmonary Aspergillosis demonstrate clathrin-mediated cellular uptake, improved antifungal efficacy and lung retention
Source: J Nanobiotechnology. 2021 Jan 11;19:19. doi: 10.1186/s12951-020-00731-1 (PMC7798018; doi:10.1186/s12951-020-00731-1)
Supplement: Supplementary file 1 — Additional file 1: Tables and figures. [file 12951_2020_731_MOESM1_ESM.docx]

**Methods:**

*Surface Activity using Langmuir Trough*

*Interaction of LPH NPs with model cell membrane monolayers*

Briefly, the cells were grown at 37°C (25 flasks, 75 cm^2^) in complete F12K medium, in the presence of carbon dioxide. At 100% confluency, the cells were detached using trypsin and centrifuged at 350 *g* for 10 minutes at 4°C. The supernatant was decanted off, the pellet was suspended in a mixture of 3 mL of chloroform and methanol (1:2), and vortexed for 5 minutes for a period of 30 minutes. An aliquot (500 µL) of this mixture was then added to distilled water (900 µL) and centrifuged (350 *g*) for 10 minutes. Dilution and centrifugation steps were repeated at least 6-7 times in order to increase the lipid purity. The remaining solvents were evaporated under the flux of nitrogen gas (0.5 to 1 psi), and the dried lipids were stored at -20°C, until used.

**Table S1.** Taguchi design matrix respective at low and high levels

| **Trials** | **CH** | **TPP** | **T80** | **ST** | **SS** | | **PST** | **pH** |
| --- | --- | --- | --- | --- | --- | --- | --- | --- |
| 1 | -1 | 1 | 1 | 1 | 1 | | -1 | -1 |
| 2 | -1 | -1 | -1 | -1 | -1 | | -1 | -1 |
| 3 | 1 | -1 | 1 | -1 | 1 | | -1 | 1 |
| 4 | 1 | 1 | -1 | 1 | -1 | | -1 | 1 |
| 5 | 1 | -1 | 1 | 1 | -1 | | 1 | -1 |
| 6 | 1 | 1 | -1 | -1 | 1 | | 1 | -1 |
| 7 | -1 | 1 | 1 | -1 | -1 | | 1 | 1 |
| 8 | -1 | -1 | -1 | 1 | 1 | | 1 | 1 |
| **Factors** | | | **Low (-1)** | | | **High (+1)** | | |
| Chitosan (CH, mg. mL^-1^) | | | 1 | | | 3 | | |
| Sodium-tripolyphosphate (TPP, mg. mL^-1^) | | | 0.5 | | | 2.5 | | |
| Tween 80 (T80, mg. mL^-1^) | | | 1 | | | 5 | | |
| Stirring time (ST, min) | | | 20 | | | 60 | | |
| Stirring speed (SS, rpm) | | | 400 | | | 1200 | | |
| Probe sonication time (min) | | | 2 | | | 5 | | |
| pH | | | 3.5 | | | 5.5 | | |

**Table S2.** Design matrix as per Box-Behnken Design

| **Formulation code** | **CRITICAL MATERIAL ATTRIBUTES (CMAs)** | | | |
| --- | --- | --- | --- | --- |
|  | **CH** | | **TPP** | **T80** |
| 1 | 0 | | -1 | -1 |
| 2 | 0 | | 1 | -1 |
| 3 | 0 | | 0 | 0 |
| 4 | 1 | | 0 | 1 |
| 5 | 1 | | -1 | 0 |
| 6 | 0 | | -1 | 1 |
| 7 | 1 | | 0 | -1 |
| 8 | 1 | | 1 | 0 |
| 9 | -1 | | 0 | -1 |
| 10 | -1 | | 1 | 0 |
| 11 | 0 | | 0 | 0 |
| 12 | -1 | | 0 | 1 |
| 13 | 0 | | 0 | 0 |
| 14 | -1 | | -1 | 0 |
| 15 | 0 | | 1 | 1 |
| 16 | 0 | | 0 | 0 |
| 17 | 0 | | 0 | 0 |
| **Factors** | | **Low (-1)** | | **High (+1)** |
|  |  |  |  |  |
| Chitosan  (CH, mg. mL^-1^) | | 1 | | 3 |
| Sodium-tripolyphosphate  (TPP, mg. mL^-1^) | | 0.5 | | 2.5 |
| Tween 80  (T80, mg. mL^-1^) | | 1 | | 5 |

**Table S3.** Coefficients of the second order quadratic polynomial model for each critical quality attributes of CH NPs

| **Model Coefficients** | **Particle**  **Size** | **Polydispersity Index** | **Zeta Potential** | **Entrapment Efficiency** | **Drug Release (8h)** |
| --- | --- | --- | --- | --- | --- |
| ß_0_ | +181.32 | +0.36 | +17.20 | +43.03 | +68.71 |
| ß_1_ | +123.07 | 0.01 | +5.71 | -0.24 | -0.85 |
| ß_2_ | -11.65 | -0.02 | -3.24 | +5.50 | -4.70 |
| ß_3_ | -29.17 | -0.09 | +0.58 | +2.80 | +1.49 |
| ß_4_ | -194.93 | -0.07 | -0.50 | +6.67 | -2.10 |
| ß_5_ | -3.37 | +0.03 | +0.48 | -0.42 | -0.46 |
| ß_6_ | +6.42 | -0.03 | +0.33 | +0.33 | -0.91 |
| ß_7_ | +121.53 | +0.03 | -0.03 | -5.07 | +0.21 |
| ß_8_ | +91.73 | +0.05 | -0.98 | -2.12 | +1.22 |
| ß_9_ | +5.78 | +0.08 | +0.10 | -3.76 | +0.92 |
| R | 0.9996 | 0.9763 | 0.9974 | 0.9826 | 0.9902 |
| p value | <0.0001 | <0.001 | <0.0001 | <0.0005 | <0.0005 |

**Table S4.** Particle size, PDI and Zeta Potential data of DPPC-modified chitosan nanoparticle formulations

| **DPPC (w/v)**  **(mg. mL^-1^)** | **PS ± SD** | **PDI ± SD** | **ZP ± SD** |
| --- | --- | --- | --- |
| CH NPs | 174.0 ± 12.4 | 0.366 ± 0.046 | 17.7 ± 0.42 |
| DPPC: CH NPs  (0.5:1) | 201.6 ± 10.3 | 0.460 ± 0.026 | 13.8 ± 0.20 |
| DPPC: CH NPs  (1:1) | 240.2 ± 12.1 | 0.283 ± 0.033 | 9.42 ± 0.33 |
| DPPC: CH NPs  (2:1) | 527.7 ± 17.2 | 0.369 ± 0.045 | 5.48 ± 0.48 |

*CH NPs: Chitosan nanoparticles; DPPC: Dipalymitoylphosphatidylcholine; PS: Particle size; PDI: Poly-dispersity index; ZP: Zeta potential; SD: Standard deviation

**Table S5.** Effect of various cryoprotectants on globule size and polydispersity index of lipid-polymer hybrid nanoparticles

| **Cryoprotectant** | **PS ± SD** | **PDI ± SD** |
| --- | --- | --- |
| 7.5% Trehalose | 299.9 ± 16.5 | 0.294 ± 0.051 |
| 10 % Trehalose | 271.6 ± 11.1 | 0.275 ± 0.016 |
| 5% Trehalose +5% Mannitol | 268.9 ± 15.6 | 0.305 ± 0.017 |
| 7.5% Mannitol + 2.5% Trehalose | 787.7 ± 20.23 | 0.829 ± 0.145 |

*PS: Particle size; PDI: Poly-dispersity index; SD: Standard deviation

**Table S6:** Mathematical drug release kinetic modelling parameters of lipid-polymer hybrid nanoparticles of VRC

| **Drug Release Model** | **R** | **Slope** | **Intercept** |
| --- | --- | --- | --- |
| Zero-order | 0.722 | 0.687 | 45.325 |
| First-order | 0.665 | 0.006 | 1.638 |
| Higuchi | 0.858 | 6.027 | 35.888 |
| Korsemeyer  Peppas | 0.927 | 0.202 | 1.559 |

1. B.

C. D.

E. F.

G. H.

**Fig. S1.** Half-normal plots and Pareto charts depicting the influence of MAs and PPs on the CQAs, (A-B) Particle size; (C-D) PDI; (E, F) Zeta potential; (G-H) Entrapment efficiency of CH NPs


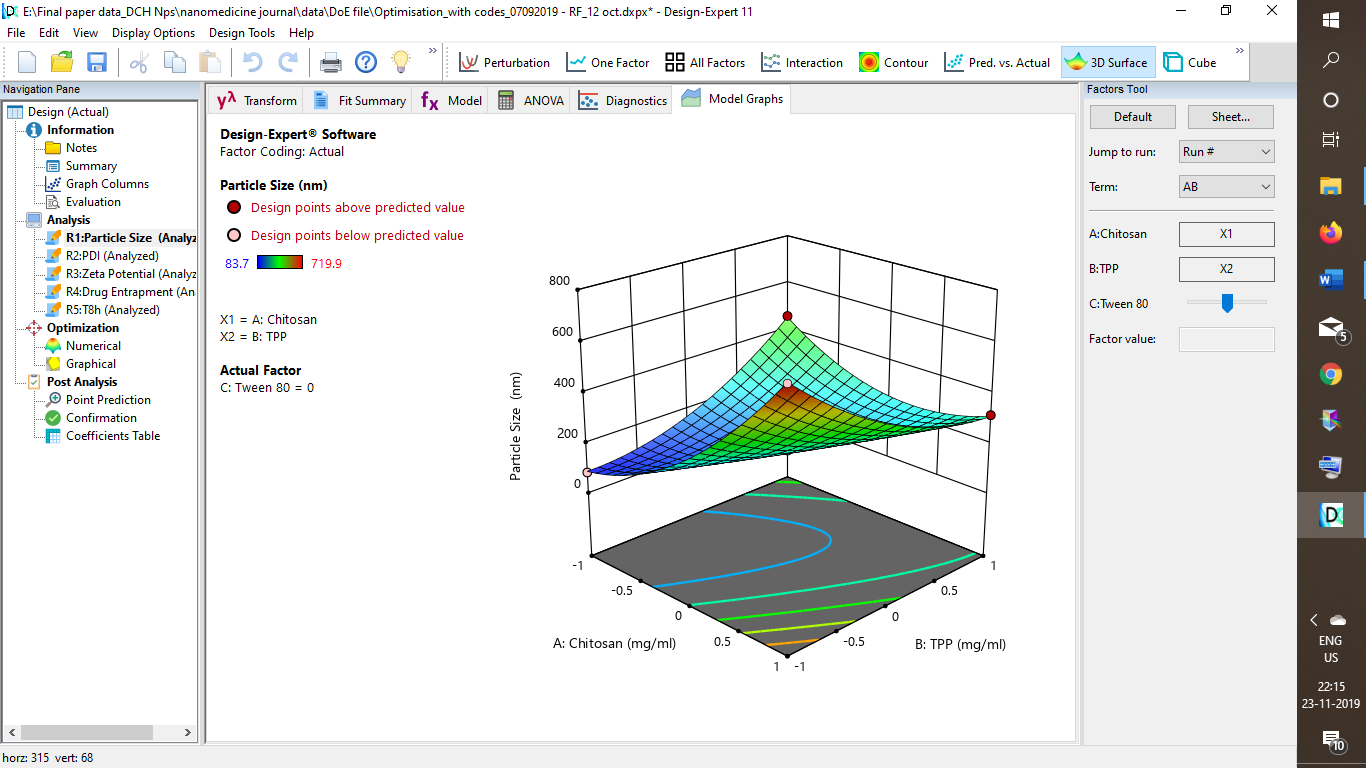

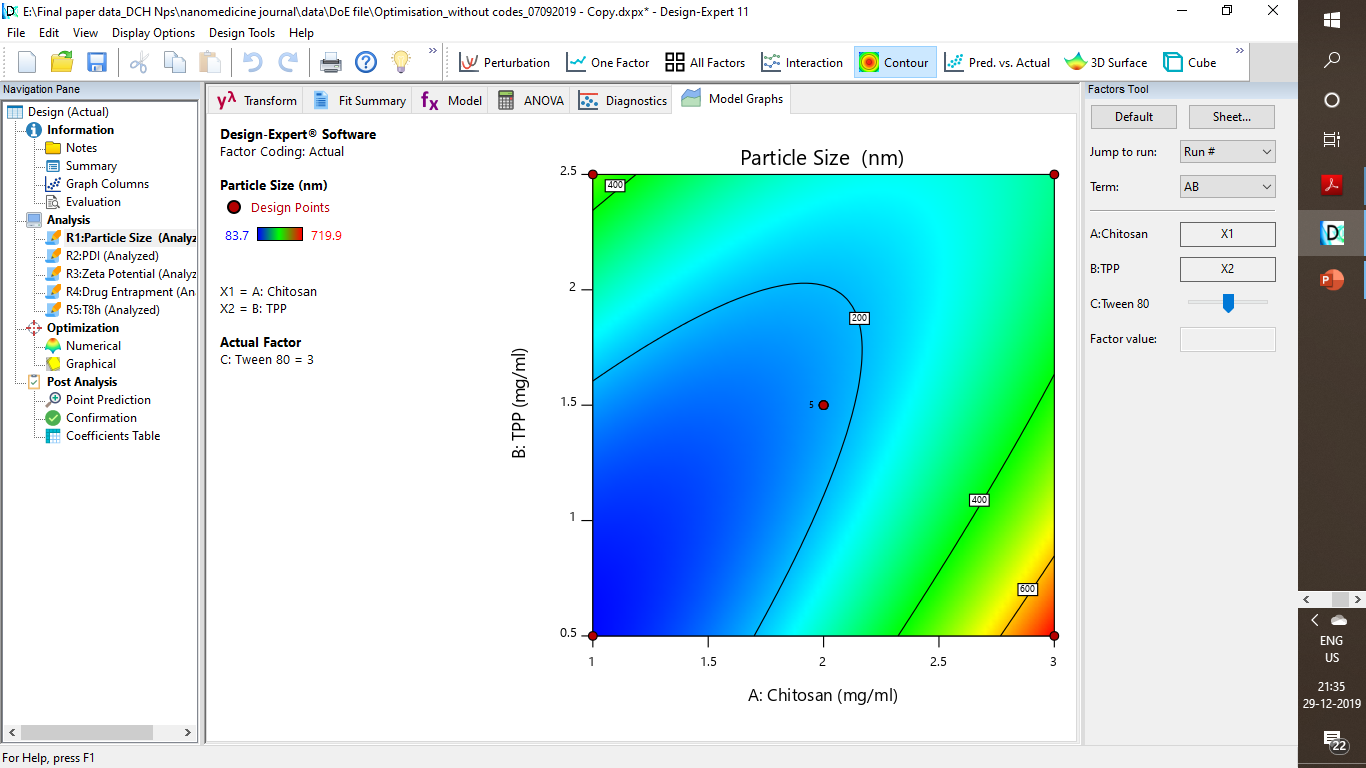


A.


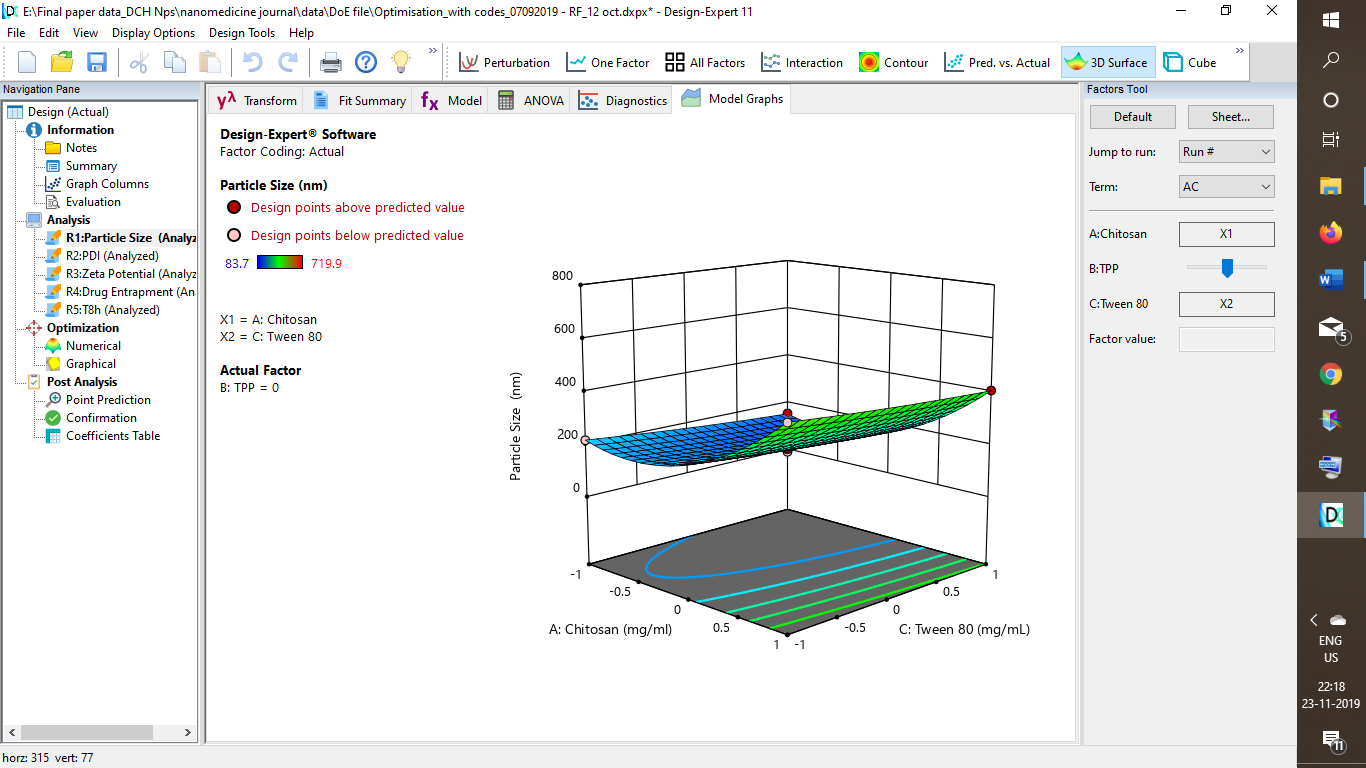


B.


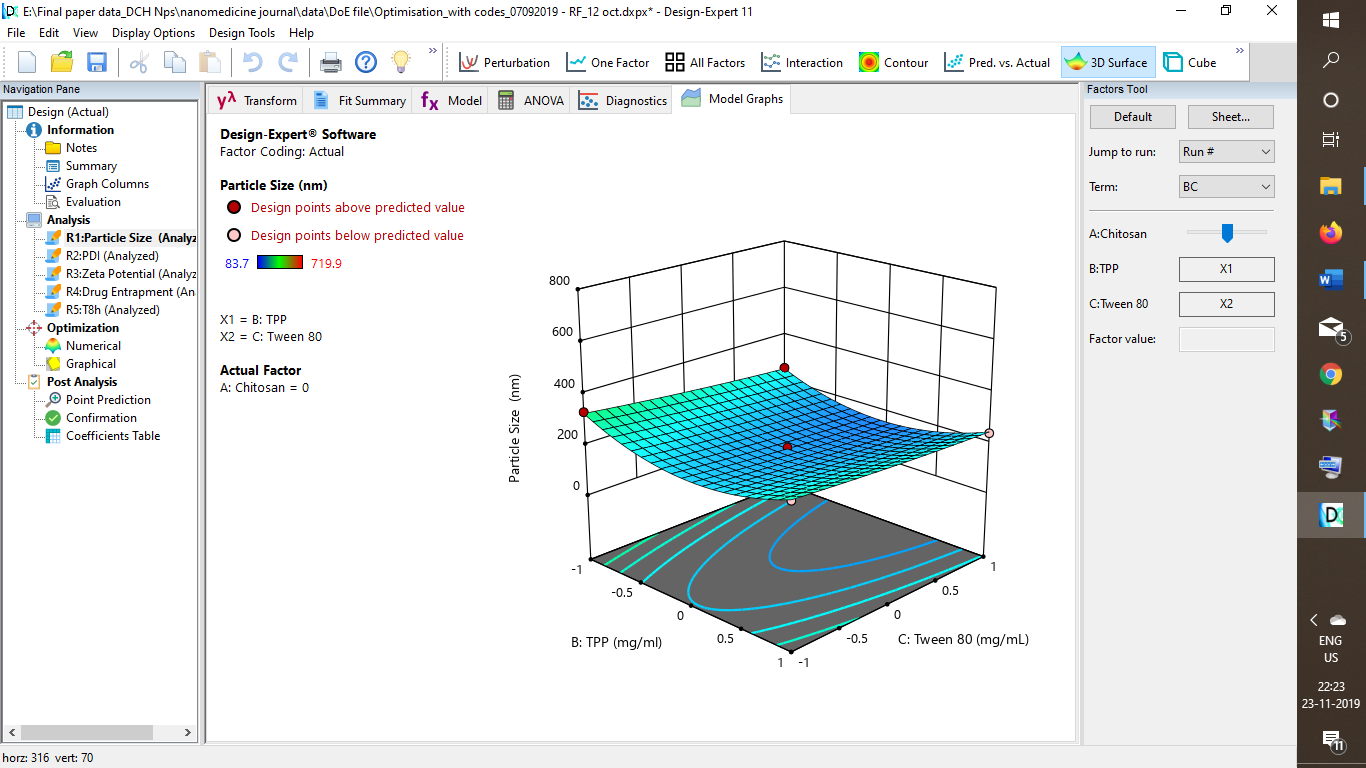


C.

**Fig. S2 (A-C).** 3D response surfaces and 2D contour plots depicting the influence of CMAs and CPPs on particle size of CH NPs


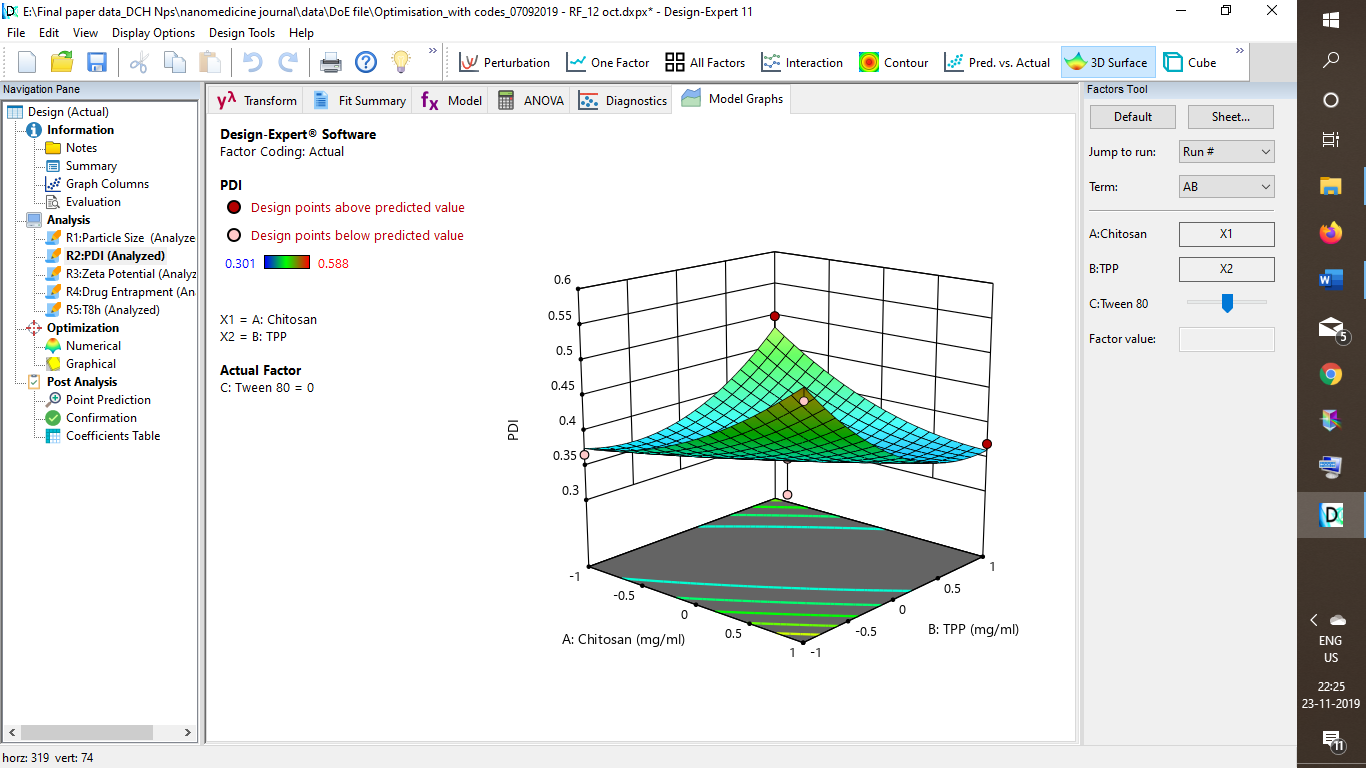


D.


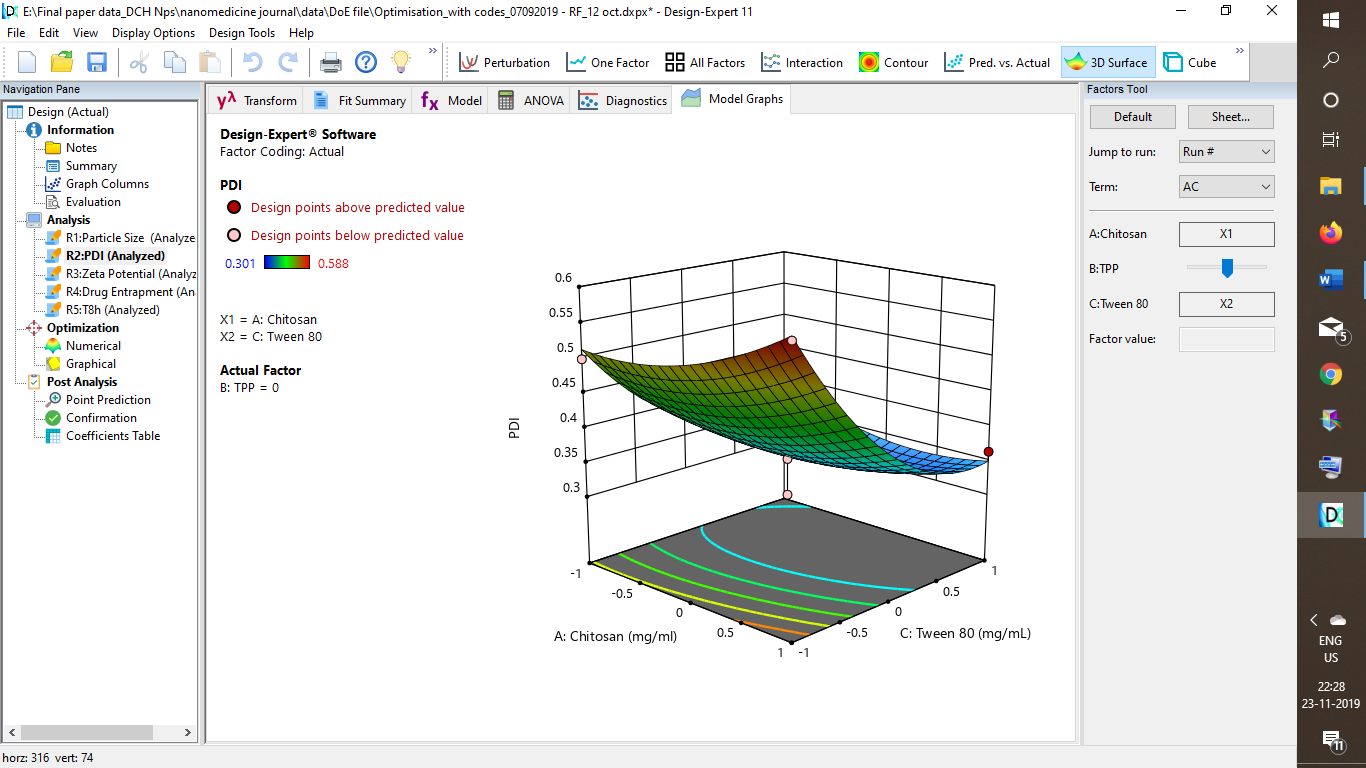


E.

D.


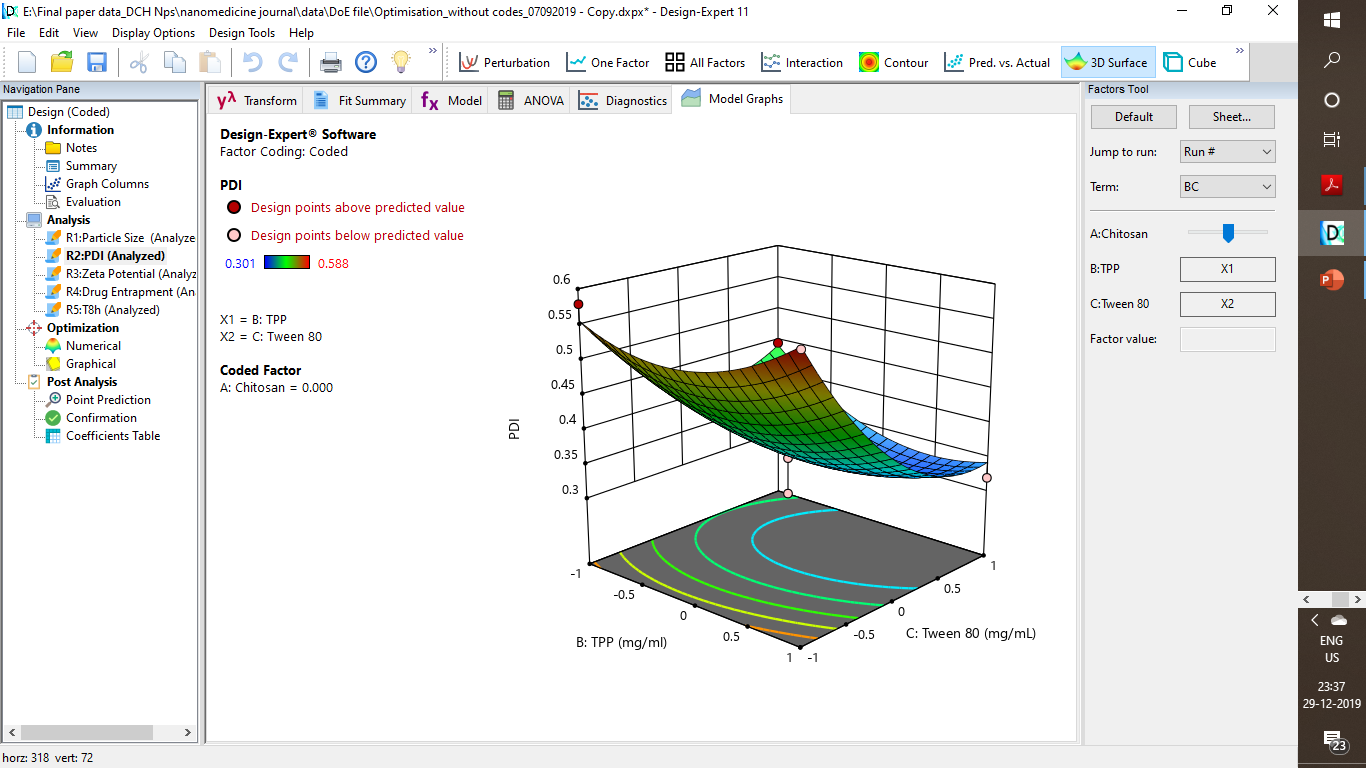

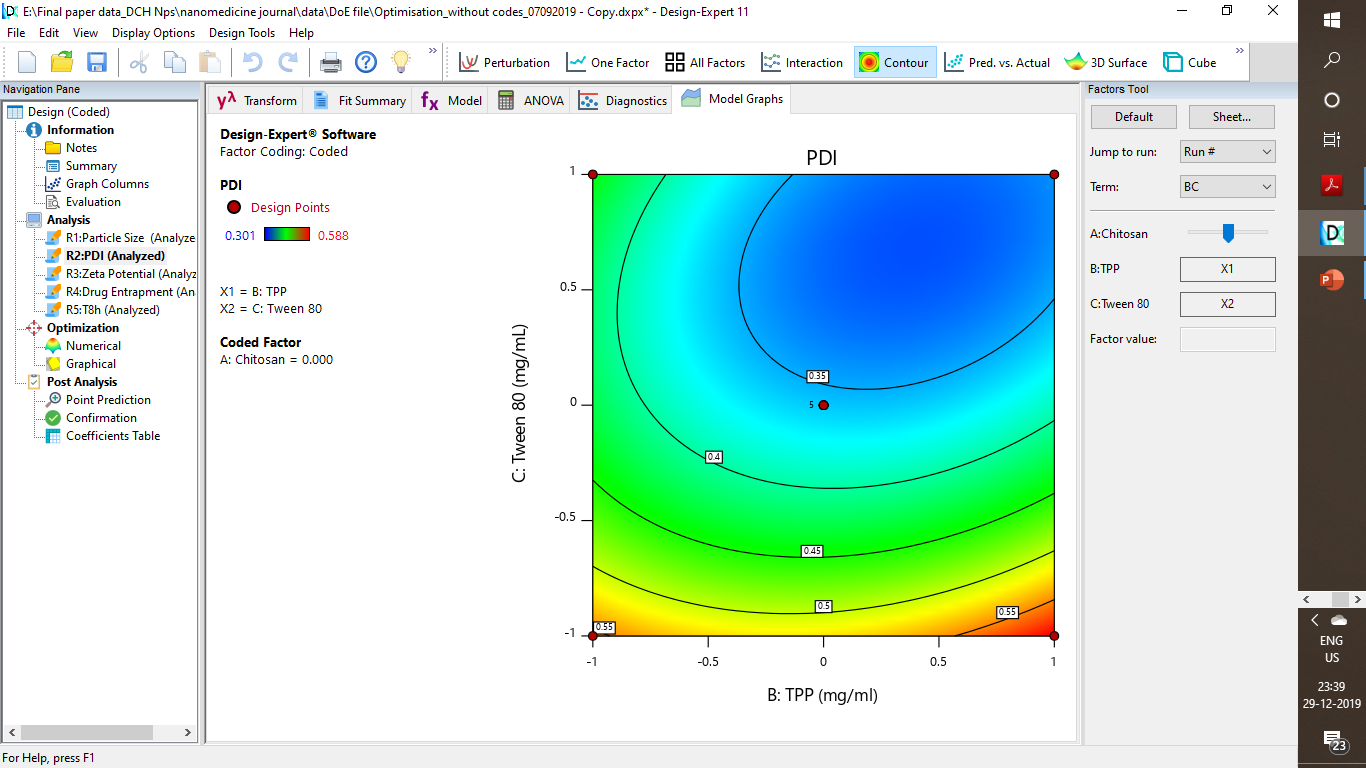


F.

**Fig. S2 (D-F).** 3D response surfaces and 2D contour plots depicting the influence of CMAs and CPPs on polydispersity index of CH NPs


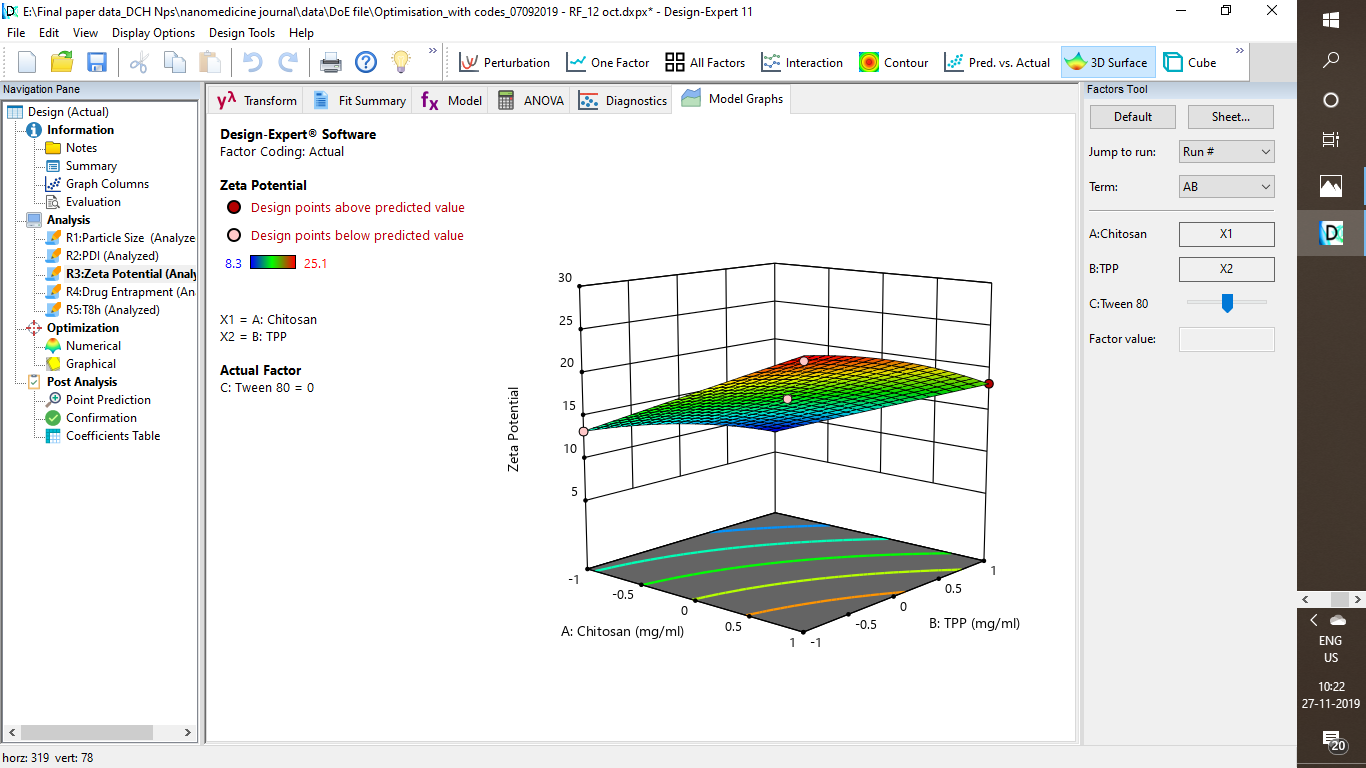


G.


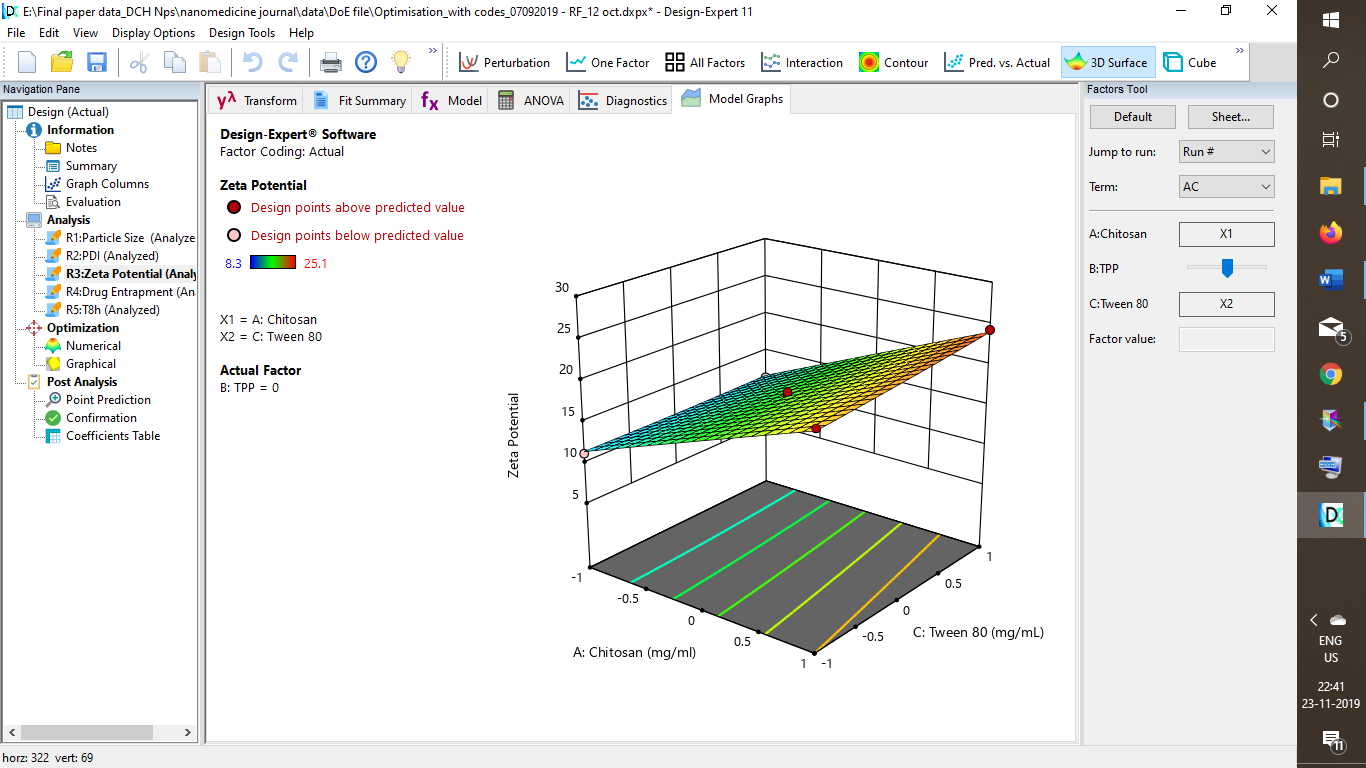


H.


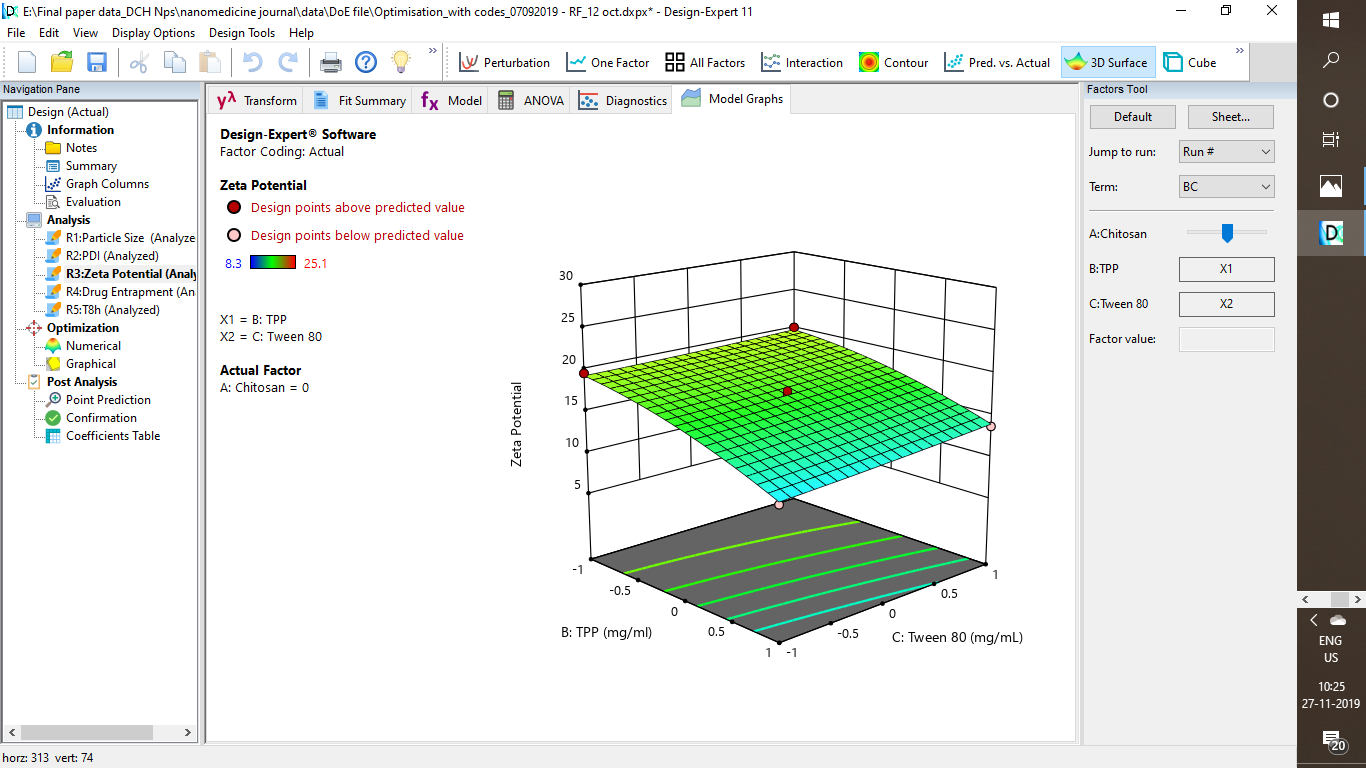


I.

**Fig. S2 (G-I).** 3D response surfaces and 2D contour plots depicting the influence of CMAs and CPPs on zeta potential of CH NPs


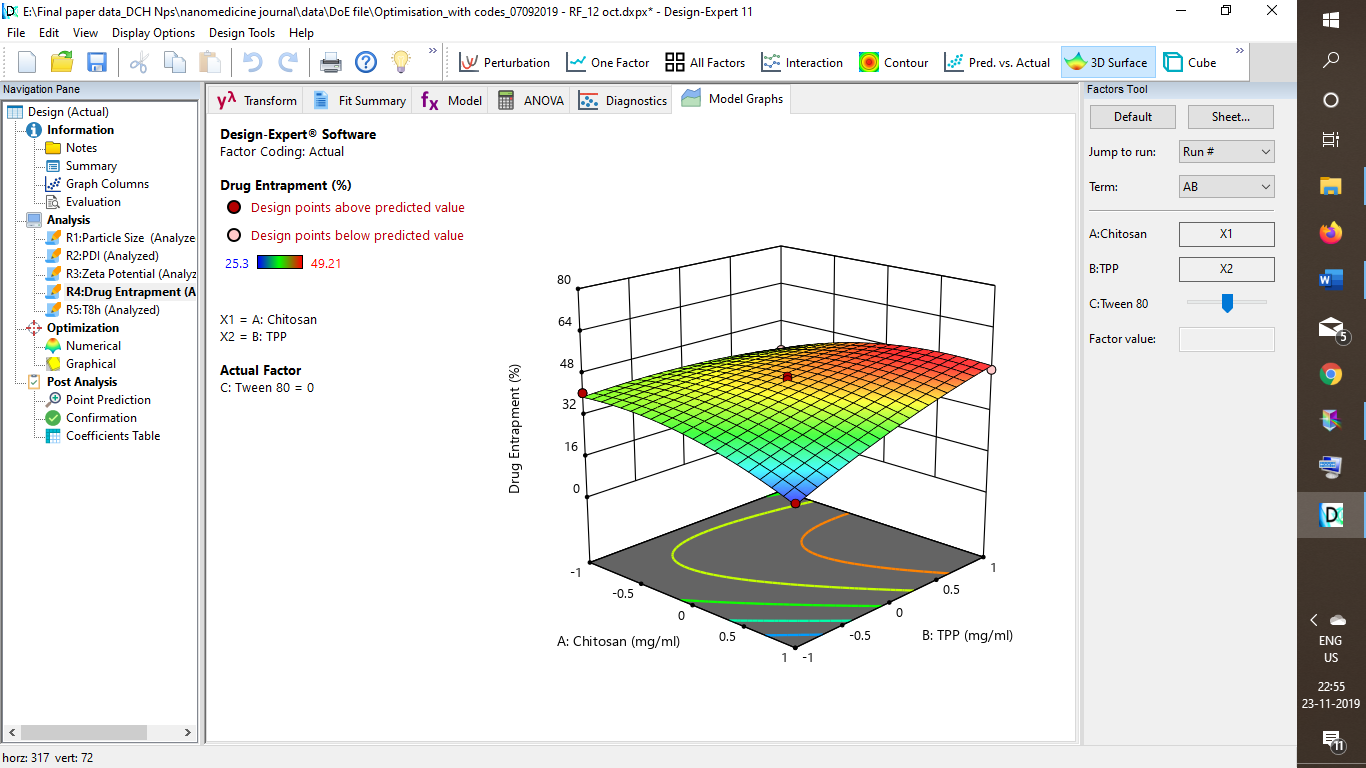


J.


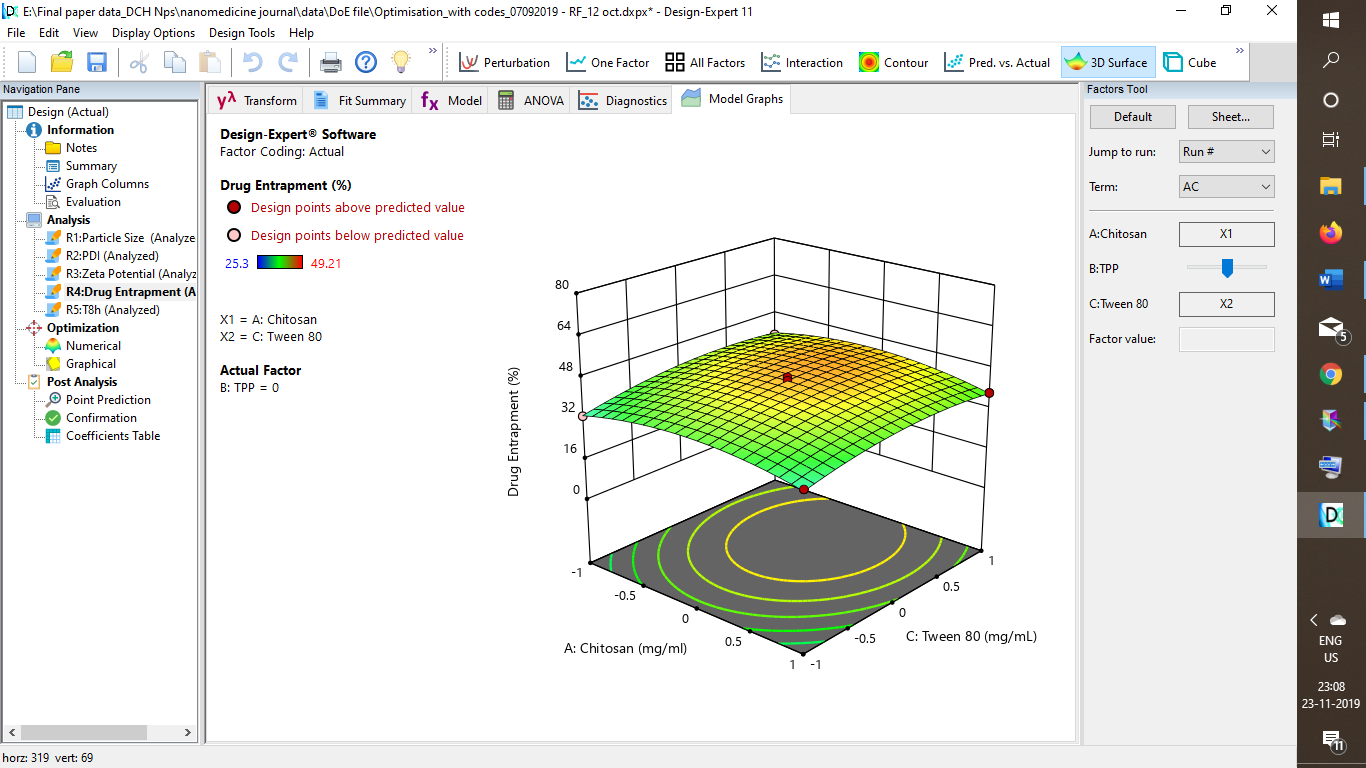


K.


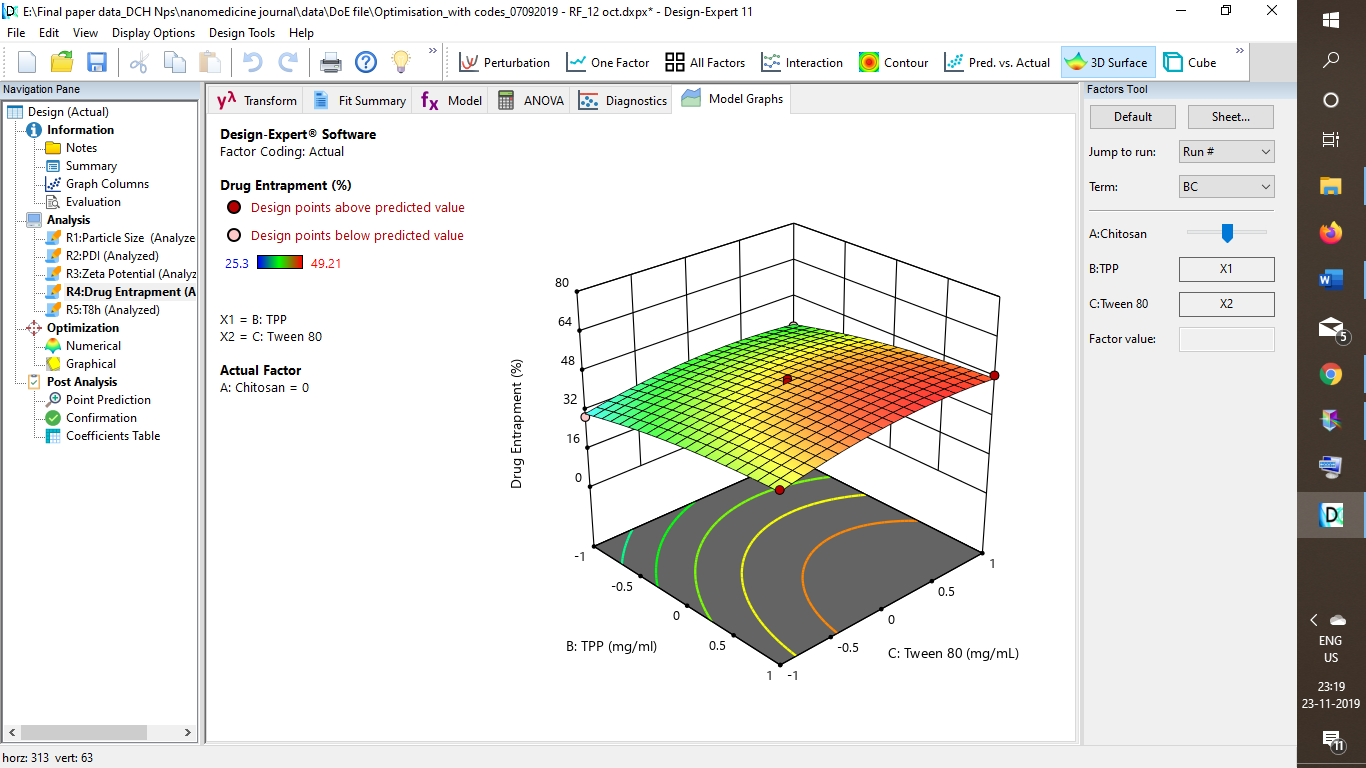


L.

**Fig. S2 (J-L).** 3D response surfaces and 2D contour plots depicting the influence of CMAs and CPPs on entrapment efficiency of CH NPs


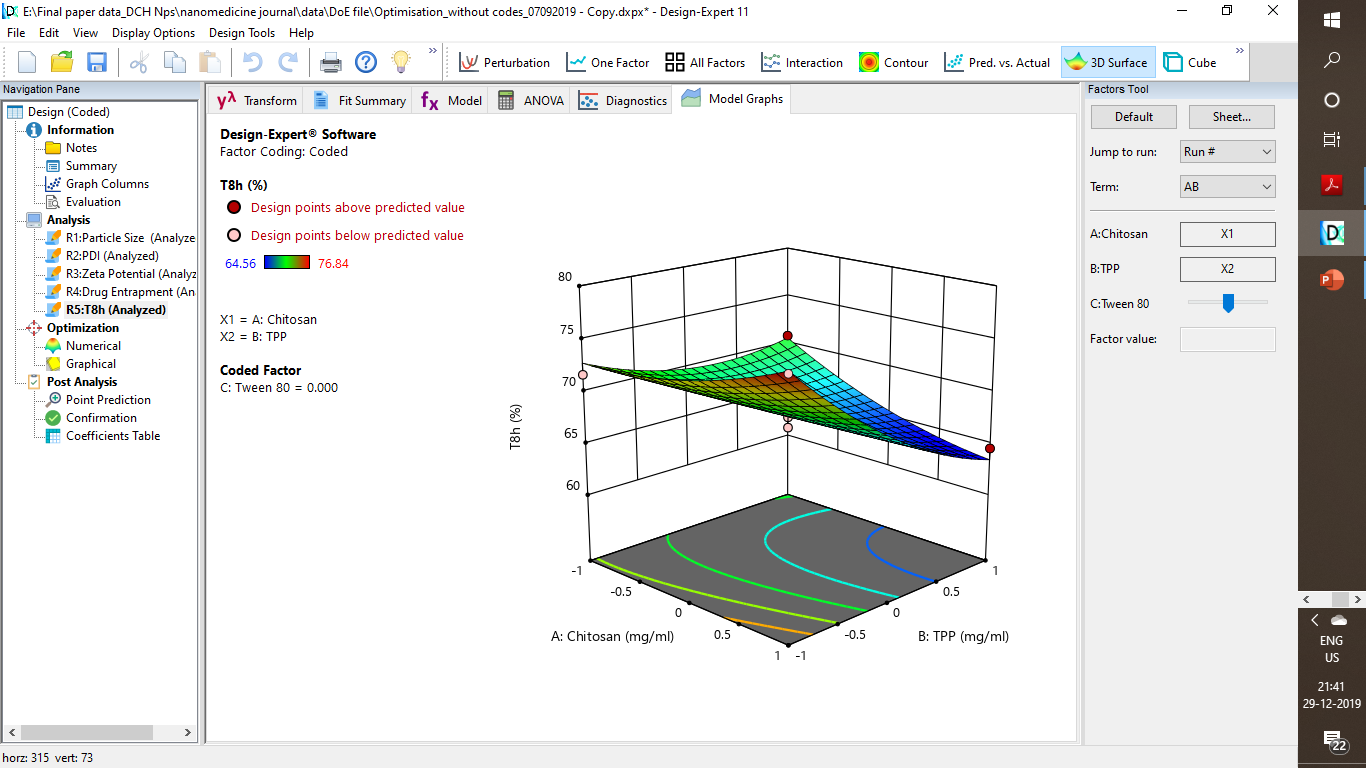

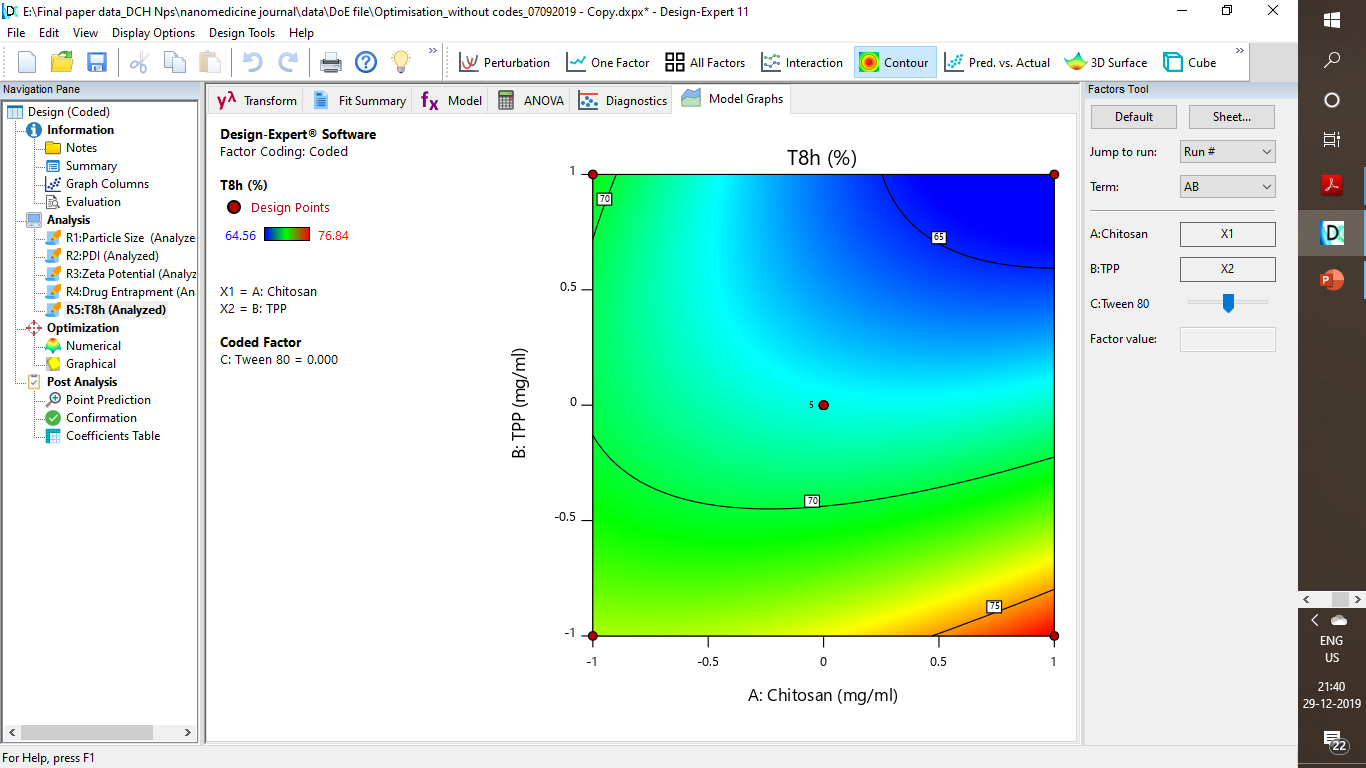


M.


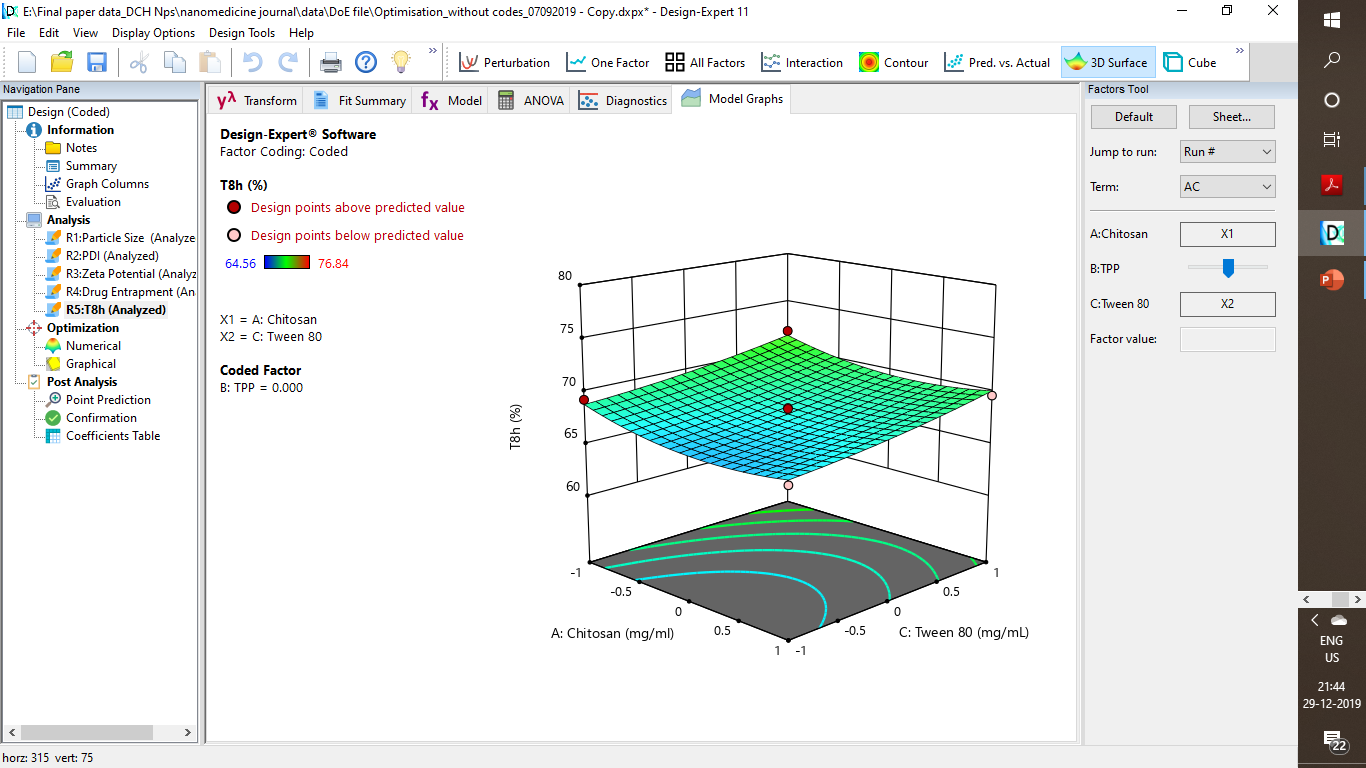

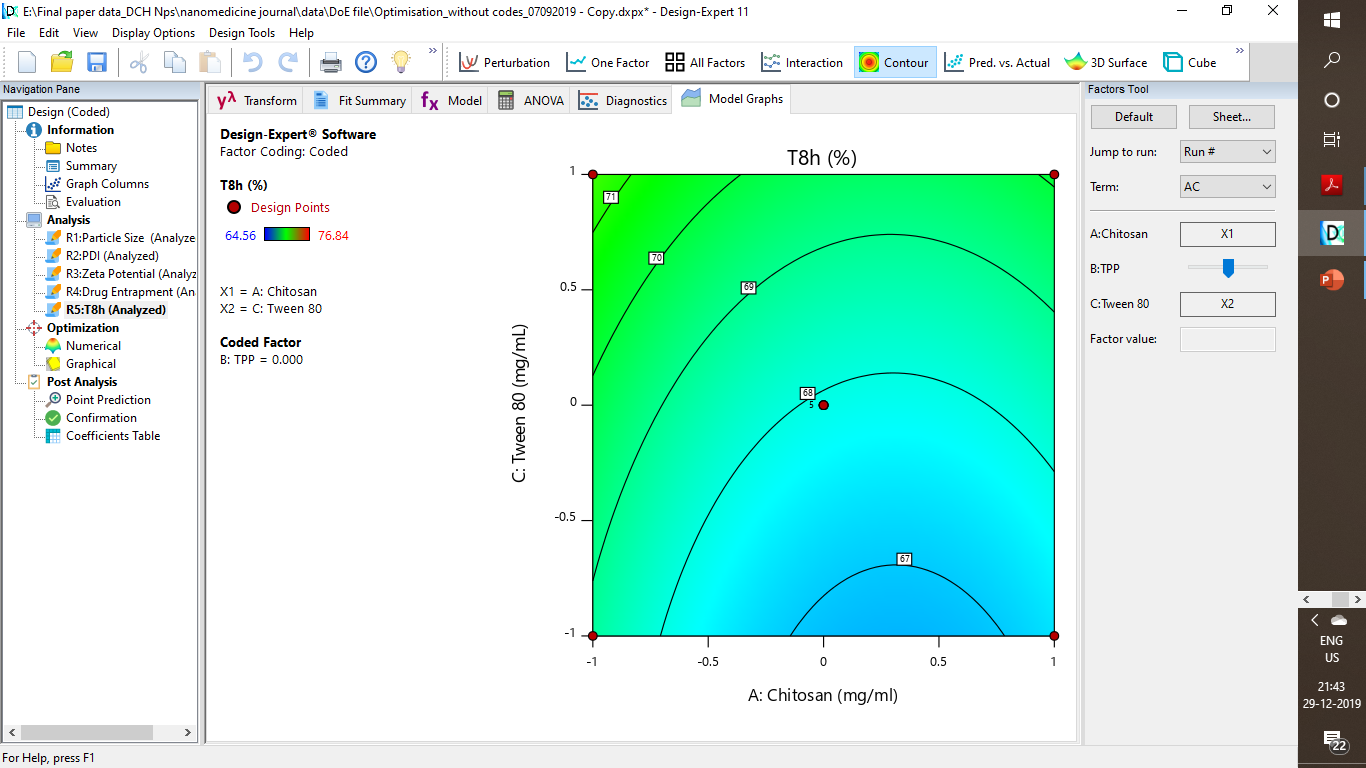


N.


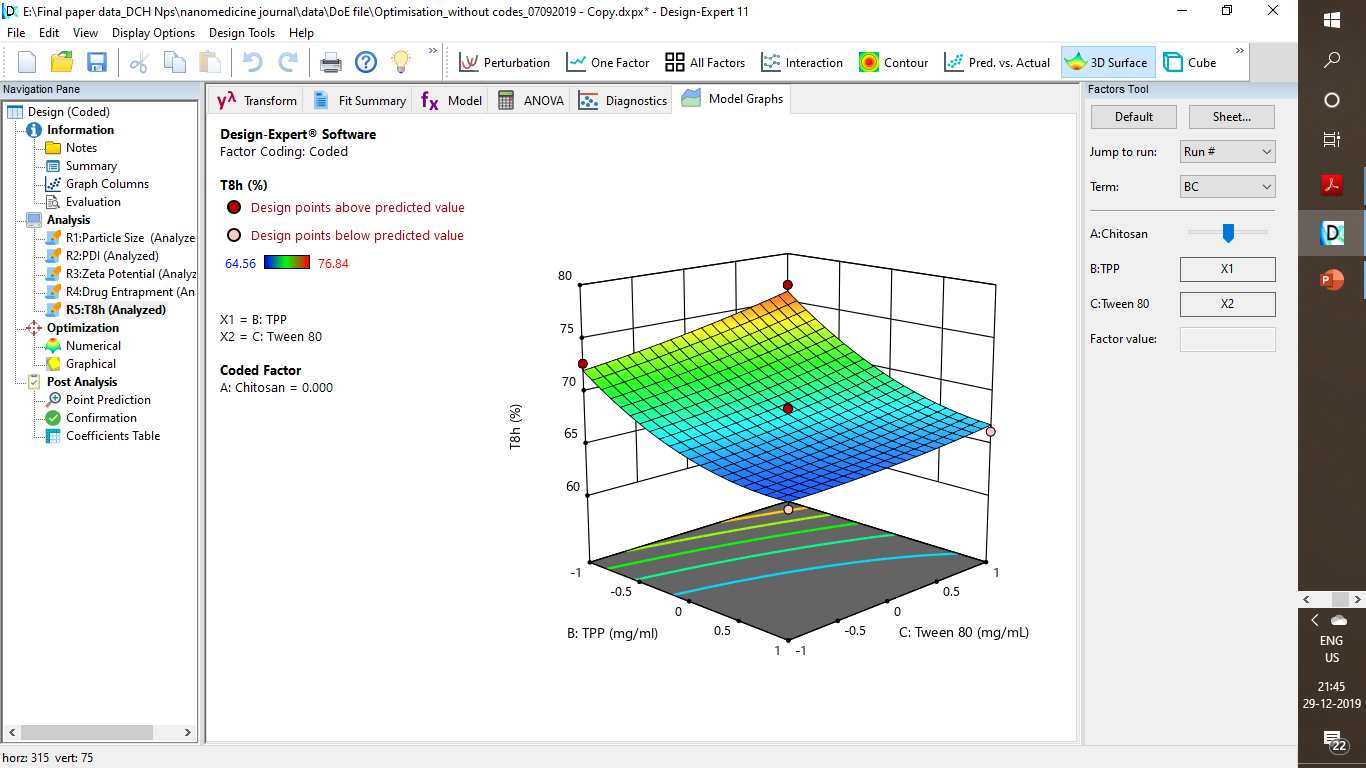

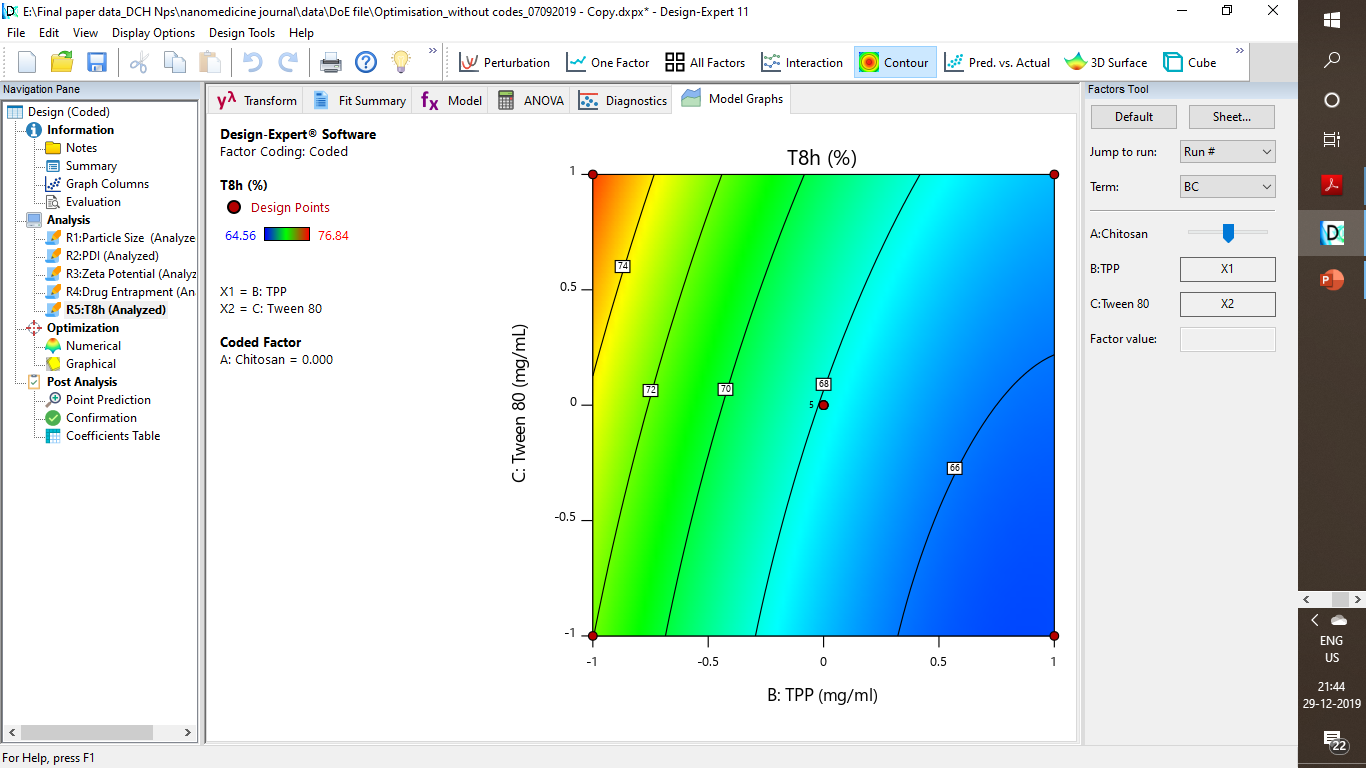


O.

**Fig. S2 (M-O).** 3D response surfaces and 2D contour plots depicting the influence of CMAs and CPPs on drug release of CH NPs at 8h


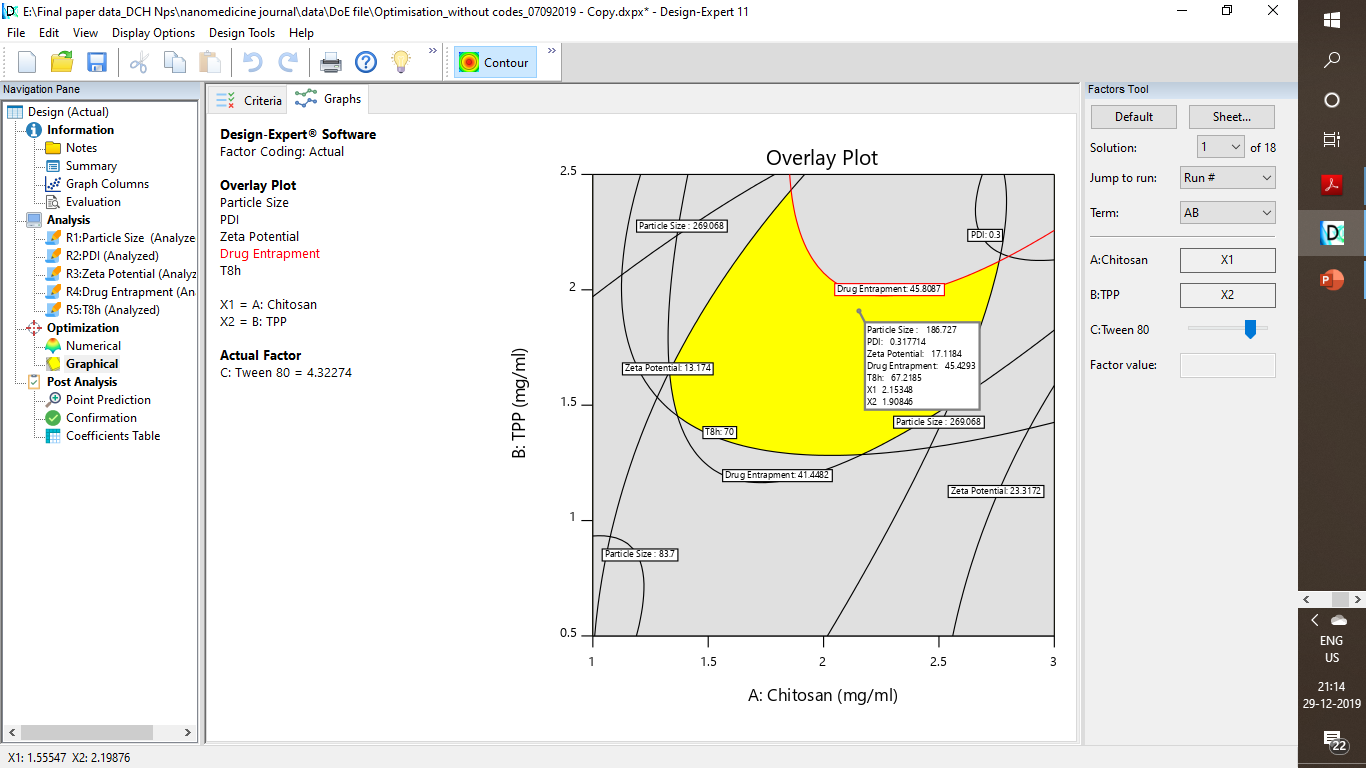

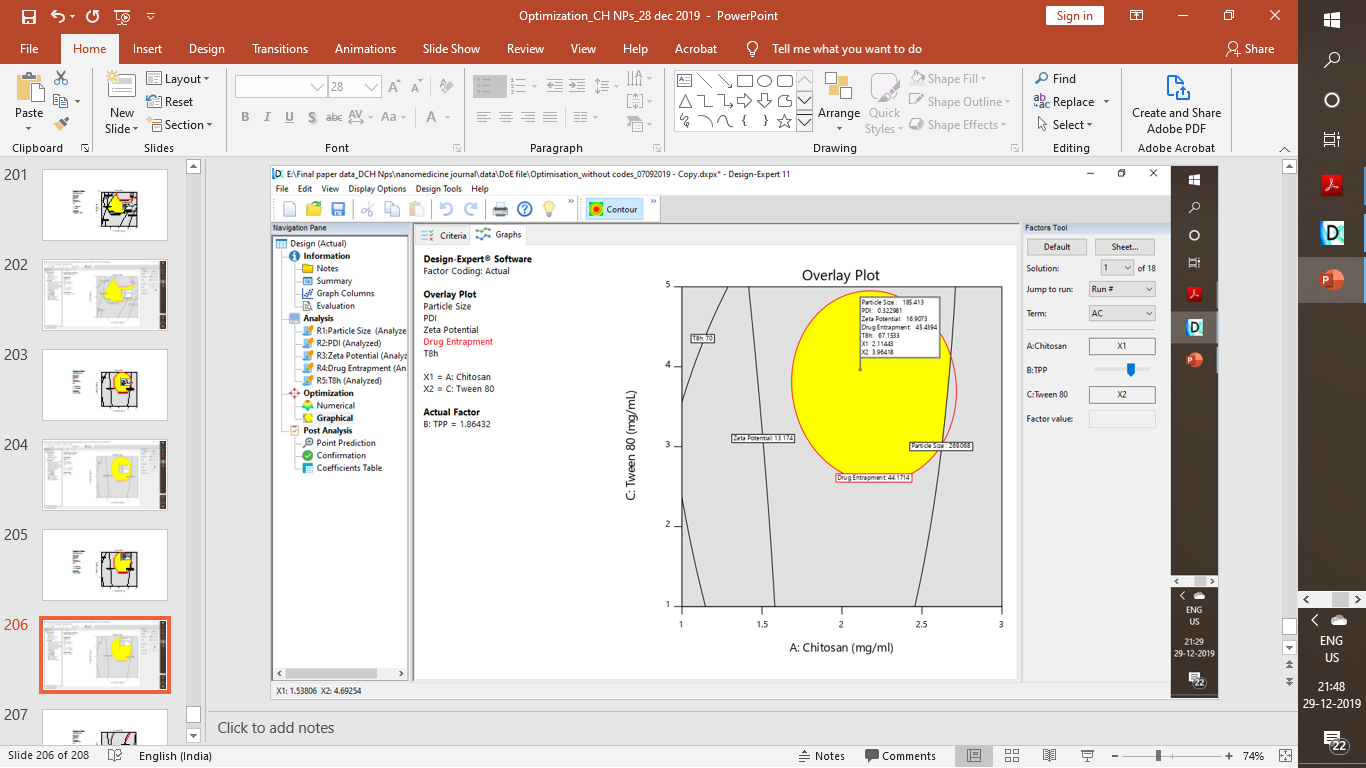


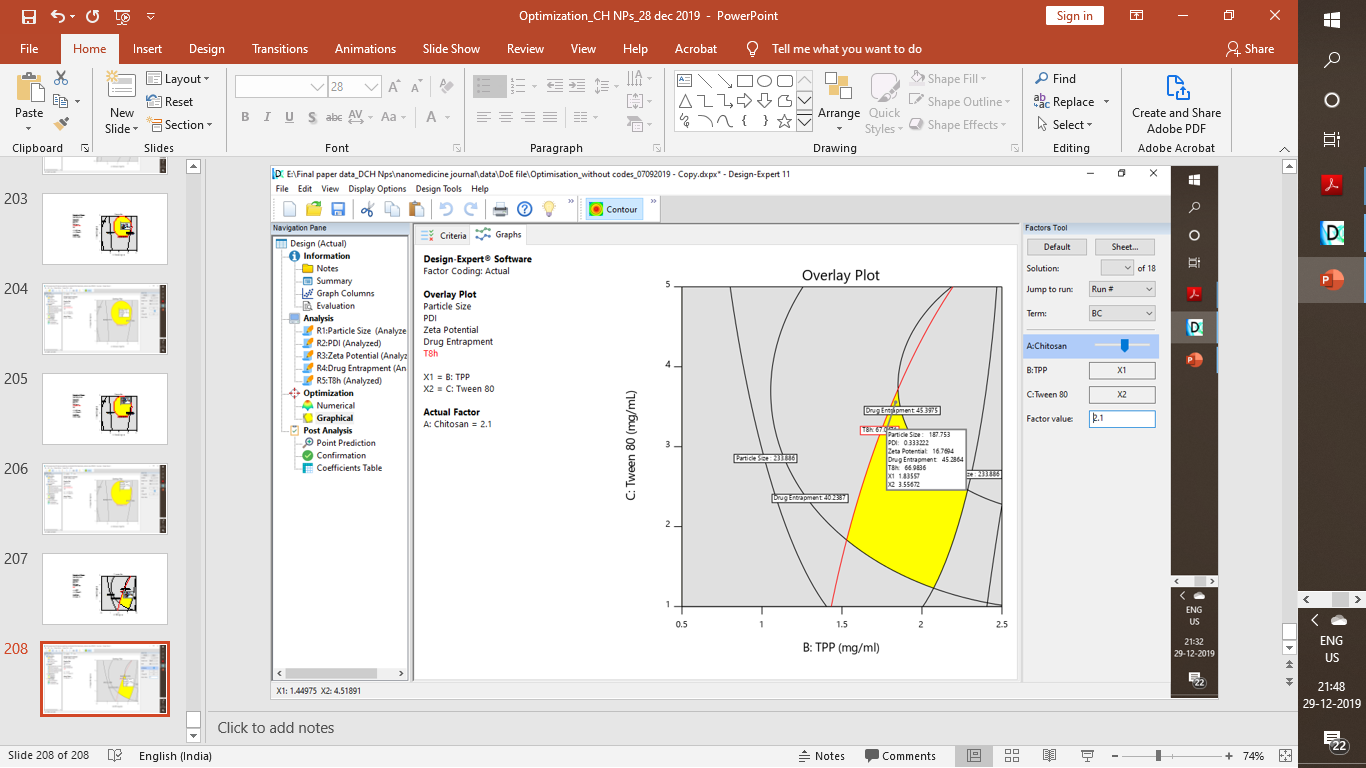


**Fig. S3**. Overlay plots portraying the design space (yellow colour), and the demarcation of the optimised CH NPs formulation


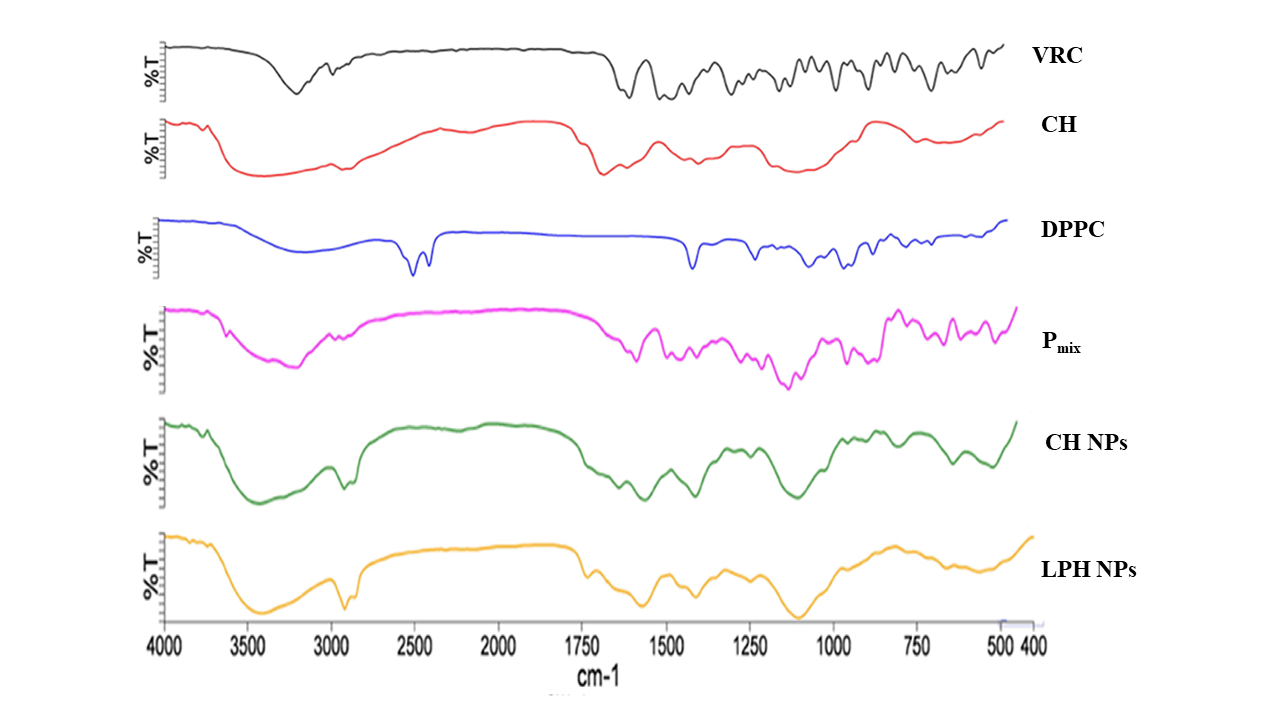


**Fig. S4**. FTIR spectra overlay of voriconazole (VRC); chitosan (CH); dipalmitoyl phosphatidylcholine (DPPC); physical mixture (P_mix_) containing drug along with CH, sodium tripolyphosphate and DPPC; chitosan nanoparticles (CH NPs) and lipid-polymer hybrid nanoparticles (LPH NPs)


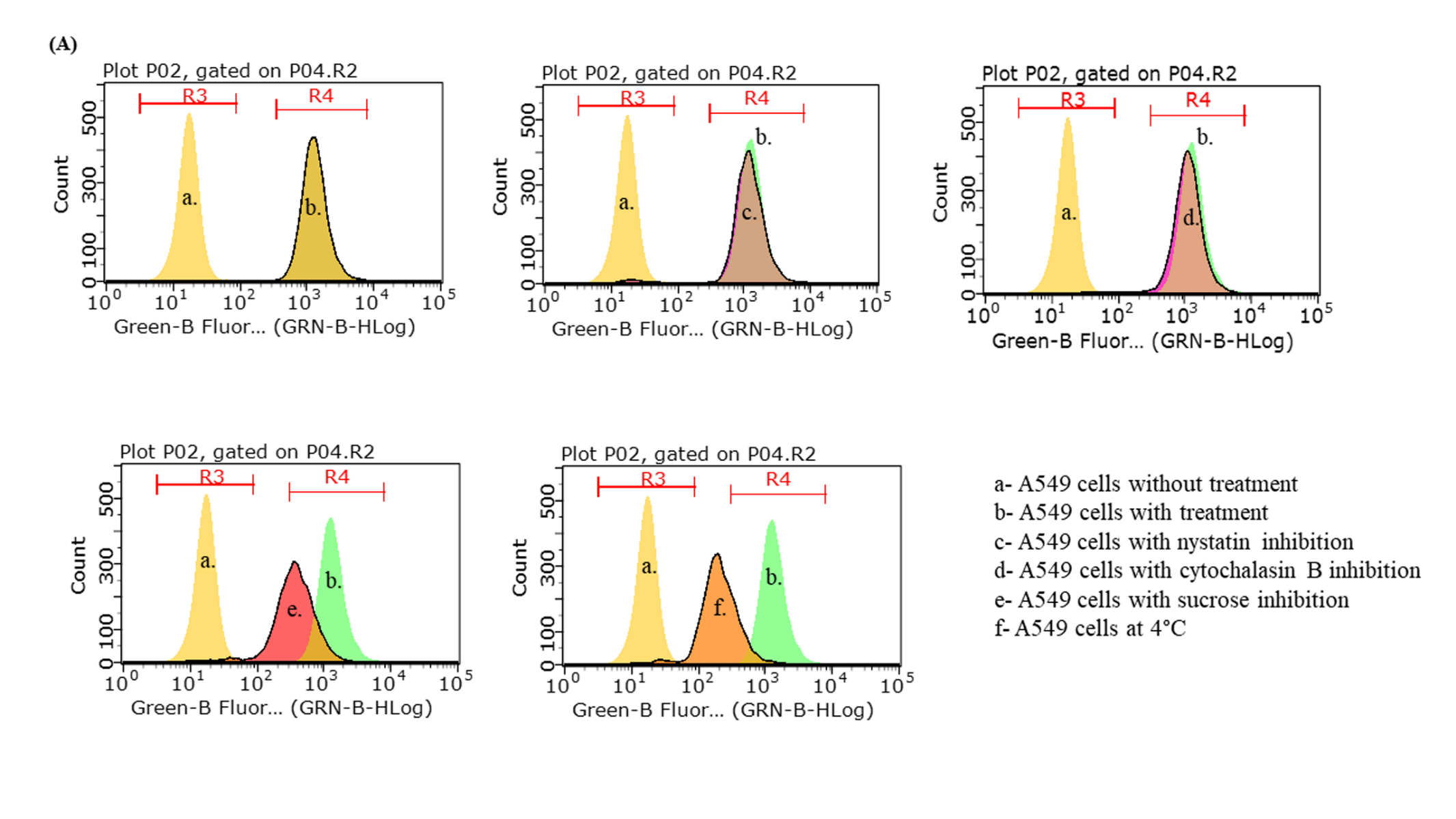


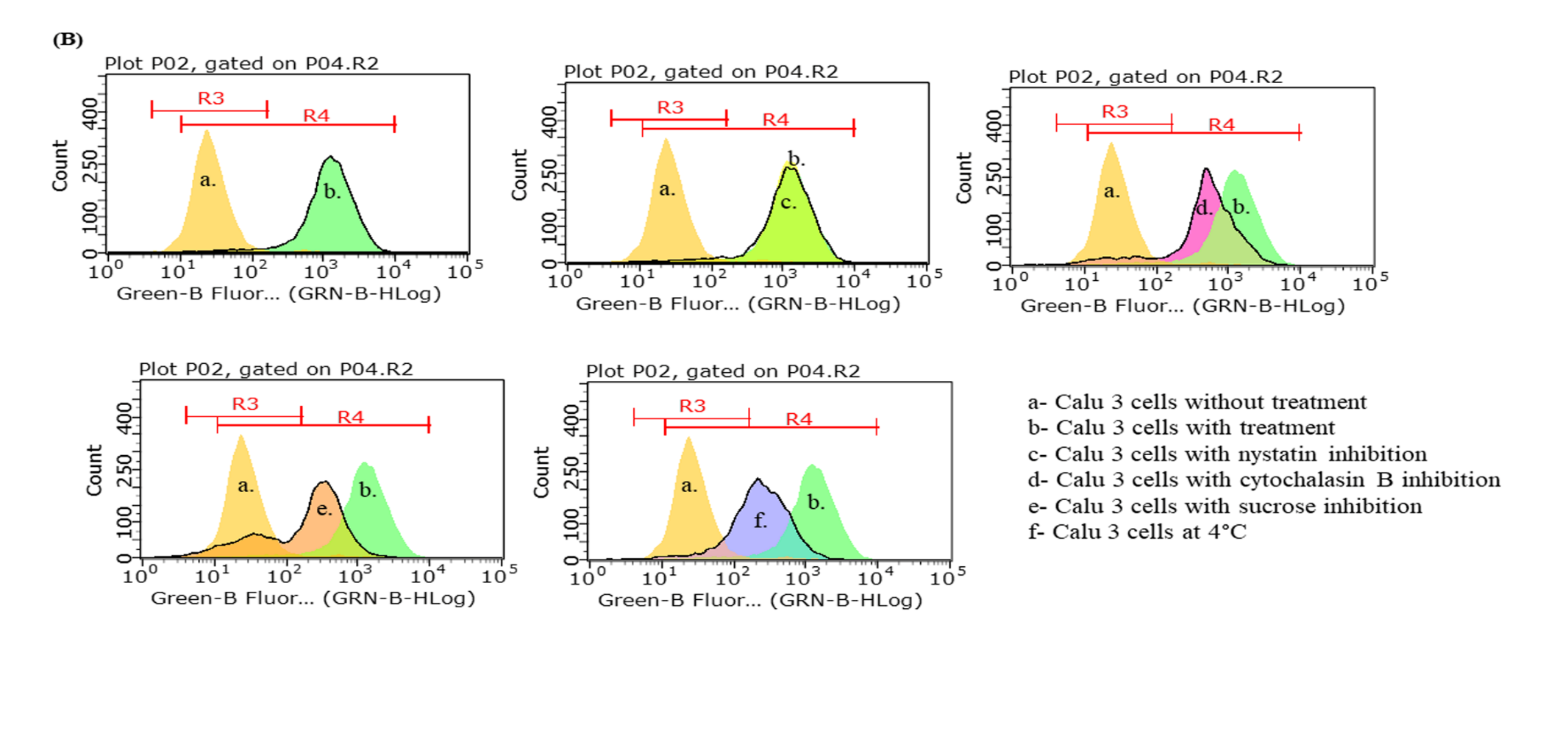


**Fig. S5.** Quantitative flow cytometer analysis to illustrate the cellular uptake of LPH NPs A. in A549 cells; and B. in Calu-3 cells


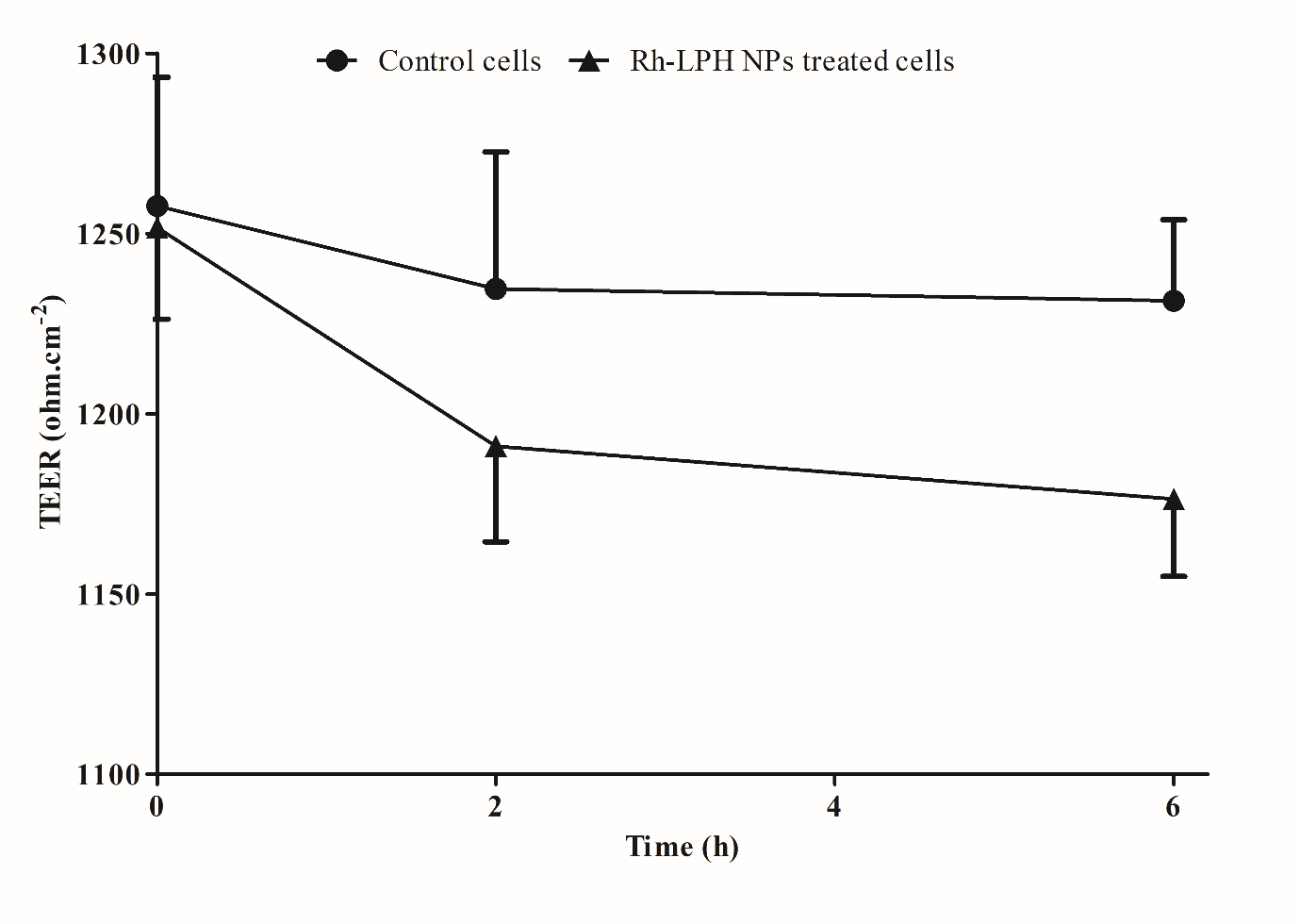


**Fig. S6:** Experimental TEER value profile of Calu 3 monolayers after treatment with rhodamine labelled LPH NPs

**Discussion of 3D-response surface and 2D contour plots**

The magnitudes of the coefficients of second-order polynomial (i.e., quadratic model) were deciphered (Supplementary **Table S3**) by Design Expert software ver. 11.0 (Stat-Ease, Inc., Minneapolis, USA) using multiple linear regression analysis (MLRA) option, as per **Equation 3**.

y = 𝜷_𝟎_ + 𝜷_𝟏_𝑿_𝟏_ + 𝜷_𝟐_𝑿_𝟐_ + 𝜷_𝟑_𝑿_3_ + 𝜷_𝟒_𝑿_1_ X_2_ + 𝜷_𝟓_𝑿_1_ X_3_+ 𝜷_𝟔_𝑿_2_ X_3_ + 𝜷_𝟕_𝑿_𝟏_^2^ + 𝜷_𝟖_𝑿_𝟐_^𝟐^+ 𝜷_9_𝑿_3_^𝟐^ **…. Equation 3**

where, y is the response variable, β_0_ represents the intercept form, β_1_ to β_3_ depict the coefficients of the corresponding linear model terms, β_4_ to β_6_ are the coefficients of interaction terms, while β_7_ to β_9_ portray the coefficients of quadratic model terms.

The response surface plots tend to facilitate explicit understanding of the studied factors, along their plausible interactions, on each of the studied CQAs of CH NPs formulation. The 3D response surface plots and 2D contour plots, constructed for each CQAs, *viz.* PS, PDI, ZP, EE, and DR, are depicted in **Fig. S2 (A-O).**

The 3D-response surface curve in **Fig. S2 A** depicts the influence of chosen CMPs, *viz.,* CH and TPP on PS of CH NPs. A nonlinear upsurge in the PS of NPs is observed at higher levels of polymer (*i.e.,* CH) and lower levels of cross-linker (*i.e.,* TPP), thereby indicating a strong interaction among the studied CMAs. With increasing levels of TPP, however, a curvilinear decline in PS is observed at intermediate to high levels. This could be ostensibly attributed to enhanced ionic gelation between the positively charged CH and negatively charged TPP. The minimum PS for CH NPs in **Fig. S2 B** is noticed at mid-levels of CH, while hardly any effect of surfactant (*i.e.,* T80) is found while in **Fig. S2 C** lower values of PS is experienced at mid-levels of TPP and high levels of surfactant. Accordingly, mid-levels of the polymer and crosslinker, and mid to high levels of surfactant are found to be conducive to obtain minimal values of PS of CH NPs with possible attainment of the zone of colloidal particles. The corresponding 2D contour plot, (**Fig. S2 A)** indicates a rising ridge pattern, *i.e,* increasing the levels of both CH and TPP tends to increase the PS, while nearly vertical lines are observed in 2D contour plot (**Fig. S2 B)**, indicating almost negligible effect of T80 on PS. **Fig. S2 C** depicts the curved nature of the contour lines with the presence of a *region of minimum (i.e., nadir)* at the centre of plot, indicating the minimum values of PS, while the extreme (*i.e.,* low and high both) levels of TPP are associated with high magnitudes of PS of CH NPs.

**Fig. S2 D** portrays an inversely proportional relationship between CH and TPP levels, *i.e.,* low values of PDI are obtained at low levels of CH and high levels of TPP, and vice-versa. **Fig. S2 E and Fig. S2 F** exhibit strong influence of T80, while modest effect of CH and TPP is observed on the PDI of CH NPs. Mid-levels of polymer, crosslinker and mid to high levels of surfactant are, therefore, found to be conducive for the formation of monodispersed CH NPs. The corresponding 2D contour plot in **Fig. S2 D** depicts stationary ridge pattern with minimal PDI at the intermediate levels of CH and TPP, while **Fig. S2 E and F** represent a curvilinear trend with almost analogous observations, *i.e.,* minimal values of PDI at mid to high levels of T80.

Maximal values of ZP for CH NPs are observed at high levels of polymer and low levels of crosslinker (**Fig. S2 G**), plausibly owing to inadequate charge neutralization of cationic polymer by the anionic crosslinker. However, T80 being non-ionic in nature, shows negligible influence on ZP of CH NPs (**Fig. S2 H** and **I)**. Identical observations can be deduced from the corresponding 2D contour plots with somewhat curvilinear and nearly vertical lines in **Fig. S2 G** and, **Fig. S2 H** and **I,** respectively.

The 3D response surface plot (**Fig. S2 J)** illustrates the relationship between polymer and crosslinker on EE of CH NPs. A linear increase in EE is observed with increasing levels of TPP, while a decline in its value is noticed with increasing CH levels. Maximal values of EE are observed at higher levels of TPP and intermediate levels of CH, attributable to efficient crosslinking of the polymer by TPP to entrap the drug molecule within the polymer-crosslinker network. A curvilinear trend is observed in EE (**Fig. S2 K**) with change in levels of both the chosen CMAs, *i.e.,* maximal EE is observed at mid-levels of CH and mid to high levels of T80. Further, **Fig. S2 L** depicts a linear inclining trend in EE, with both the CMA’s, *i.e.,* maximum values of EE for CH NPs are observed at high levels of crosslinker and surfactant. The corresponding 2D contour plot (**Fig. S2 J)** illustrates a rising ridge pattern, while **Fig. S2 K** represents a concentric pattern with maximal value of EE observed at the intermediate levels of polymer and intermediate to high levels of surfactant. Also, **Fig. S2 L** depicts a curvilinear trend with maximal values of EE at the high levels of TPP. The presence of curved response surface confirms the prevalence of interactions among the studied factors on the values of EE.

The 3D response surface plot (**Fig. S2 M)** represents minimum value of VRC release (T8h) from CH NPs at the intermediate levels of polymer and the high levels of crosslinker**.** However, modest linear increase in T8h (**Fig. S2 N)** is observed, with increase in levels of T80 while a linear declining pattern (**Fig. S2 O)** is observed with increasing the TPP levels. Likewise, the 2D contour plots deciphered attainment of lower values of T8h at mid-levels of polymer and surfactant, coupled with mid to high levels of the crosslinker.
